# A Study of Non-Linear Manifold Feature Extraction in Spike Sorting

**DOI:** 10.1007/s12021-025-09744-3

**Published:** 2025-10-02

**Authors:** Eugen-Richard Ardelean, Raluca Portase

**Affiliations:** Department of Computer Science, Technical University of Cluj-Napoca, Cluj-Napoca, Romania

**Keywords:** Feature extraction, Non-linear, Manifold, Spike sorting, Neuroscience

## Abstract

With recent developments in recording hardware, the processing of neuronal data must keep up with the increasing volumes and complexity by capturing the intrinsic relationships between instances of neuronal activity while remaining invariant to noise. Here, we explore a suite of non-linear manifold feature extraction methods – including PHATE, t-SNE, UMAP, TriMap – in an attempt to identify the most adequate method for automated spike sorting. Spike sorting is the process of clustering instances of neuronal activity, called spikes, based on similarity. By embedding high-dimensional spike shapes into low-dimensional manifolds that preserve local and global structure, we demonstrate more separable and robust clusters than those obtained via traditional feature extraction methods, such as PCA. We evaluated all feature extraction methods analyzed on 95 single-channel synthetic datasets and 2 single-channel real datasets spanning a range of cluster counts. Quantitative evaluation using clustering performance metrics (such as Adjusted Rand Index, Silhouette Score, etc.) indicates that several manifold feature extractions outperform other feature extraction methods. Our results suggest that the embeddings obtained by non-linear manifold approaches can offer a powerful, high-precision option in the spike sorting of the next-generation of electrophysiological recordings. While this study focuses on single-channel data and a subset of manifold learning techniques, a baseline has been established, and future avenues of research have been opened through this work. Future work may extend these insights to multi-channel settings, such as high-density probes and incorporate emerging manifold methods, such as hierarchical and multi-view extensions, which could further improve the robustness and accuracy of spike sorting.

**Supplementary Information:**

The online version contains supplementary material available at https://doi.org/10.1007/s12021-025-09744-3.

## Introduction

### Spike Sorting

Extracellular recordings capture the neural activity as voltage fluctuations from multiple nearby neurons (Carter & Shieh, 2015), producing a continuous signal. Each individual activity of a single neuron is called a spike and in the case of extracellular recordings the neuron that generated such an activity is unknown. Spike sorting is the process of assigning each detected spike waveform (Bear et al., 2015) from an extracellular recording to its source neuron, based on the assumption that each neuron produces spikes of consistent shape (Bear et al., 2015), while different neurons generate distinguishable shapes from each other (Quiroga, 2007). However, the shape of spikes can be distorted by noise, electrode drift (Steinmetz et al., 2021) and biological variability, which result in scattered clusters instead of pinpoints. Consequently, it is important to find a robust approach to reduce the impact of these phenomena. One approach is to extract a small set of discriminative features that preserve the information that differentiates between the shapes of spikes to improve clustering.

The canonical approach to spike sorting (Buccino et al., 2022; Quiroga, 2007; Rey et al., 2015) is a pipeline of four sequential steps: filtering, spike detection and clustering. Filtering is done in a band-pass manner and is applied to the raw recorded signal to isolate the frequency band where spiking occurs (Rey et al., 2015). Spikes are typically detected through a simple amplitude thresholding based on the standard deviation of the signal multiplied by a scalar value (typically, from 3 to 5) to identify the events that could represent the neuronal activity. The choice of this threshold is a compromise between identifying noise segments as spikes and losing spikes of lower amplitude. Feature extraction is utilised to both generate a more auspicious space and to improve clustering efficiency. The newly generated feature space should be robust as in invariant to small changes in shape, providing an easily separable space for clustering (Tolas et al., 2023), while simultaneously reducing the dimensionality to improve the execution time of clustering. As the final step of the spike sorting pipeline, the spikes should be grouped to represent all instances of activity of each individual neuron. In the traditional spike sorting pipelines, the clustering algorithm does this final assignment of spikes by source neurons.

The spike sorting pipeline (Buccino et al., 2022; Rey et al., 2015) has seen many forms over the years. Initially, a supervised manual approach was taken where an expert would classify spikes based on a visual interpretation of similarity in a low-dimensional space. This reduced space was generated by simple features (Chung et al., 2017; Meister et al., 1994) such as amplitude, width, and the peak-to-trough ratio. Using the peak-to-trough ratio feature was found to be useful in determining the type of neuron as narrow spikes (small peak-to-trough ratio) are representative of inhibitory neurons, while excitatory neurons have wider spikes (Ebbesen et al., 2016). Since the 1950 s, the number of recorded neurons has increased exponentially (Stevenson & Kording, 2011), rendering manual approaches unfeasible, and recent developments in recording hardware (Jun et al., 2017; Steinmetz et al., 2021) follow this trend. Through empirical analysis, probabilistic models were created that were able to leverage the entire spike waveform (Pouzat et al., 2002), allowing for the processing of a low number of electrodes. Later, the high-dimensional space of the spike waveform was projected to lower-dimensional spaces by applying PCA (Litke et al., 2004) and time–frequency transforms (such as the Wavelet Transform (Hulata et al., 2002)) have started being used to introduce the frequency information in the computation.

The choice of approach also depends upon the nature of the analysis, whether offline or online. Offline spike sorting allows for the use of more complex algorithms as there is no time constraint as the analysis is done after the recording has finished. However, in an online setting, the algorithms employed must have the ability to process the data during the recording, thus requiring low execution times.

A common approach taken lately is template matching (Pachitariu et al., 2016; Pachitariu et al., 2024) on subsets of data. Usually, this approach substitutes the steps of spike detection, feature extraction, and even clustering in the canonical spike sorting pipeline. Due to its application to subsets rather than the whole data, it is an efficient approach from a computational perspective. One such algorithm that employs template matching is M-Sorter (Yuan et al., 2012). M-Sorter is an automated approach to spike detection and classification based on coefficients obtained through the wavelet transform and template matching. This method can be seen as a two-stage process when applied to the filtered signal. It employs correlation of the wavelet coefficients for the detection of the spikes, while through the use of K-Means templates are generated which are used in the assignment of spikes to the neurons that produced them (considered to be that which has the smallest distance). Another template matching-based approach to spike sorting is Kilosort (Pachitariu et al., 2016; Pachitariu et al., 2024) which combines spike detection and clustering into a process called template learning. Kilosort was developed for handling high-density probes (Steinmetz et al., 2021), such as Neuropixels, yet it has been shown to be performant for other types of probes as well (Pachitariu et al., 2024). Kilosort also employs an iterative matching pursuit step to effectively detect and resolve overlapping spikes. Kilosort4 further enhances performance by integrating drift correction and a graph-based clustering algorithm that includes a merging tree strategy.

In this work, we attempt to examine the impact of feature extraction in spike sorting. Although clustering outputs the final result and separation of the space into clusters, it is actually the feature extraction that must obtain a separable space for the clustering. Similarly to clustering algorithms, a golden standard (Estivill-Castro, 2002; Pedreira et al., 2012) does not exist for feature extraction algorithms (Quiroga, 2007; Rey et al., 2015) either. Their performance depends on the particular set of characteristics of the input data. Here, we employ a number of non-linear feature extraction algorithms to identify the most adequate algorithm for the spike sorting problem.

### Non-Linear Feature Extraction

The classical techniques for dimensionality reduction, such as PCA and MDS, are computationally efficient and perfectly able to find the structure of linear spaces (Analysis & [Internet], 1979). However, they encounter difficulties when non-linear structures are present (Bear et al., 2015). Non-linear manifold learning algorithms seek to discover a low-dimensional embedding (or a manifold) within the high-dimensional input data. These methods can preserve the intrinsic geometry (including local neighborhood and data topology) by approximating the underlying manifold, rather than relying on global linear projections such as PCA (Adamos et al., 2008; Mishra et al., 2017).

Each detected spike waveform can be viewed as a high-dimensional vector in spike sorting. We can consider that the shapes of spikes vary from their ‘true shape’ due to recording artefacts. Therefore, non-linear manifold feature extraction techniques may disentangle these factors by yielding robust embeddings to perturbations (Belkin & Niyogi, 2003) and offer separability in overlapping clusters (generated by linear techniques). Moreover, modern manifold techniques have been designed to handle large volumes of data by employing sparse neighborhood graphs and optimization for scalability (Amid & TriMap, 2022; McInnes et al., 2020). This makes them a viable candidate (Amid & TriMap, 2022; McInnes et al., 2020) for the spike sorting of high-density probes (Steinmetz et al., 2021).

### The Challenges of Spike Sorting

Spike sorting is fundamentally complex for several reasons (Ardelean et al., 2023a). Brain recordings are inherently subject to the distortion of the spike waveforms due to the reasons specified above; these phenomena affecting spike shape generate clusters that do not have a well-defined separation boundary. This overlap of clusters is a struggle for most clustering algorithms, especially if coupled with data imbalance. Cluster imbalance in neuronal data appears from the variability in the firing rate of neurons. Neuronal activity is dynamically modulated by neural circuits, causing individual cells to fire at widely varying rates (Buzsáki, 2006; Lewicki, 1998); this variability generates clusters of disparate sizes and yields an intrinsic imbalance in the dataset. Electrode drift (Lefebvre et al., 2016; Steinmetz et al., 2021) manifests as gradual changes in the recorded waveform due to electrode/tissue movement. Multiple neurons can be active at the same time resulting in overlapping spikes, called spike collisions. Neurons may also fire multiple times in a short period of time (Ardelean et al., 2023b) with varying waveform shapes and amplitudes; this phenomenon is called bursting (Bakkum et al., 2014). Moreover, neuronal activity takes place on a millisecond timescale, thus even relatively short brain recording sessions can produce a vast quantity of data (Bear et al., 2015). In this context, single-unit activity refers to the spikes of one neuron that can be isolated as a single cluster. In contrast, spikes from more distant neurons typically appear with lower amplitudes (poor signal-to-noise ratio), and cannot be reliably separated (resulting in a single cluster being identified)—these are generally referred to as multiunit activity (Rey et al., 2015).

These non-linear manifold feature extraction methods often outperform linear feature spaces (Meilă & Zhang, 2023) and may be able to simultaneously denoise waveforms, which can create dense clusters and increase the variability between the spikes of different neurons, which can create separable clusters. In this study, we therefore evaluate a suite of representative non-linear feature extractors (e.g., Isomap, LLE, Spectral Embedding, Diffusion Maps, UMAP, TriMap) in comparison with traditional feature extraction methods and other non-linear feature extraction methods, to systematically compare how each manifold embedding influences cluster separability and spike-sorting performance across datasets.

The paper is structured as follows. Section 2 reviews traditional feature‐extraction techniques and their performance in spike sorting, outlines the proposed methods, and describes the datasets and evaluation metrics. In Sect. 3, we assess the methods across multiple metrics and offer a critical interpretation of their performance. Finally, Sect. 4 examines the limitations of the methods proposed for spike sorting and presents our concluding findings.

## Materials and Methods

### Feature Extraction Algorithms

One of the most important steps of the spike sorting pipeline is the feature extraction, where the high-dimensional space of the spike waveform is projected to a usually lower-dimensional space, which contains the most informative features. The purpose is thus dual, to preserve as much as possible from the data structure of the original feature space in the reduced space and simultaneously reduce the space as much as possible. There are many criteria by which feature extraction methods may be categorized, such as convexity or linearity (Dimensionality reduction: a comparative review., 2022) Here, we separate the methods used into 3 categories: linear, non-linear, and non-linear manifold feature extraction methods. Linear dimensionality reduction methods assume data lie near a flat, low-dimensional subspace and use linear projections to uncover that structure. Non-linear methods allow for arbitrary transformations but do not necessarily assume an underlying manifold. Manifold learning techniques are a subclass of non-linear methods that explicitly consider that the data lies on a low-dimensional manifold embedded in high-dimensional space and attempt to recover its geometry by preserving local or global relationships.

### Linear Feature Extraction Methods

The most common algorithm for feature extraction is Principal Component Analysis (PCA) (Mishra et al., 2017), and it has been thoroughly used in spike sorting (Adamos et al., 2008; Rey et al., 2015) as well. Even recently developed spike sorting pipelines employ PCA in their computations (Toosi et al., 2021). PCA identifies orthogonal directions, or eigenvectors, based on maximum variance. PCA projects the original feature space into a new feature space, called principal components, based on the eigenvectors obtained through the eigendecomposition. Essentially, PCA rotates the coordinate system to align with maximum variance. Dimensionality reduction can be achieved by discarding components while preserving data variance, most commonly only the first two or three principal components represent 70–80% of the variance of the original feature space, and only these are kept (Abeles & Goldstein, 1977; Glaser & Marks, 1968). However, variance may not be the best approach for the separability of clusters (Quiroga, 2007; Rey et al., 2015), as the discarded low-variance features may encode more information for separability.

Multidimensional Scaling (MDS) (Borg et al., 2005) creates low-dimensional representations that attempt to preserve the relationship between data points. Its classical version computes a distance matrix between all points to find coordinates in a lower-dimensional space that best match the original distances by minimising a stress function. By preserving original distances, it can be considered a linear approach. For the Euclidean distance, MDS produces results similar to those of PCA.

Independent Component Analysis (ICA) (Hyvärinen, 2013) was designed to separate multivariate signals into independent components. Nevertheless, it was also shown to be highly performant in the spike sorting domain (Lopes et al., 2013; Tiganj & Mboup, 2012). In contrast to PCA, which finds uncorrelated components, ICA seeks statistically independent sources by iteratively maximizing non-Gaussianity (using measures like kurtosis or negentropy). Thus, ICA works under the assumption that the signals are linear mixtures of non-Gaussian independent signals. The ICA algorithm effectively unmixes the signals by finding an unmixing matrix that produces the most statistically independent outputs.

#### Non-Linear Feature Extraction Methods

Kernel PCA (KPCA) (Schölkopf et al., 1997) is a non-linear extension of PCA through the use of the “kernel trick”. A non-linear kernel is utilized to map the input data into a possibly higher-dimensional feature space, followed by PCA. Through the computation and the extraction of the eigenvectors of the kernel matrix (representing the inner product space), KPCA can capture non-linear relationships that PCA misses without the additional computation of coordinates in the higher-dimensional space.

A non-metric version of Multidimensional Scaling (MDS) (Borg et al., 2005) can preserve the ordering of distances rather than the values themselves. In other words, points closer than others in the original space are also closer in the embeddings obtained. This is achieved by transforming the original space using a monotonic function and iteratively minimising the same stress function. Through the ordering of distances, the non-metric MDS may be able to preserve the structure of data points when the relationship between similarity and distance is non-linear.

Self-Organizing Map (SOM) (Ardelean et al., 2023c; Kohonen, 1982) creates a mapping between the data points and a two-dimensional grid where similar high-dimensional inputs are located nearby to each other. The grid of “neurons” is initialised in the low-dimensional space, followed by a training process where the input data is presented repeatedly to update the neuron (and the neighborhood) that best matches the input. The competitive learning process of SOMs preserves the topology of the input data.

Autoencoders (AE) (Ardelean et al., 2023; Baldi, 2012; Pinaya et al., 2019) are a type of neural network that are able to learn embeddings on the input data through an unsupervised approach. They are formed out of two sub-models, an encoder and a decoder. The encoder maps the input data to a latent embedding, while the decoder attempts to reconstruct the input data at the output. By optimizing the reconstruction, the autoencoder manages to obtain a relevant low-dimensional representation of the input.

#### Non-Linear Manifold Feature Extraction Methods

Locally Linear Embedding (LLE) (Roweis & Saul, 2000) preserves the local structure of data points by representing each as a weighted combination of its neighbours. It operates on the assumption that each neighbourhood of points lies close on a locally linear patch of the manifold. The three steps of LLE are: identifying the k-nearest neighbours of each point, computing the weights that best reconstruct each point based on its neighbours (by solving linear equations) and finding a low-dimensional representation that preserves the reconstruction weights (by solving an eigenvalue problem).

Modified Locally Linear Embedding (MLLE) (Zhang & Wang, 2006) is an extension of LLE which employs multiple weight vectors for each data point that obtain valid reconstructions. By employing alignment techniques, it allows for the identification of a global embedding that respects the constraints of each set of weight vectors.

Hessian-based Locally Linear Embedding (HLLE) (Donoho & Grimes, 2003) is another extension of LLE which use the Hessian operator to capture the local structure of the data. The Hessian matrix (representing the second derivative of the manifold) is computed for each neighbourhood of the nearest neighbours. HLLE identifies directions along which the manifold is locally flat by finding the null space of the Hessian. These directions form the basis for the low-dimensional embedding.

Local Tangent Space Alignment (LTSA) (Zhang & Zha, 2002) is another extension of LLE, which aligns local tangent spaces to capture the global structure of the data. The tangent space is computed (as a linear approximation using principal components) for neighbourhoods as the k-nearest neighbours. The embedding is found by aligning these tangent spaces (by solving an eigenvalue problem).

Isometric Mapping, or Isomap (Tenenbaum et al., 2000), attempts to maintain the geodesic distance between the data. Essentially, it flattens the manifold structure while preserving the geodesic distance. It constructs a graph connecting nodes to their nearest neighbours. A distance matrix is computed by computing the shortest paths between pairs of points/nodes. It concludes by applying MDS to obtain the low-dimensional space.

T-distributed Stochastic Neighbor Embedding (t-SNE) (Zhou et al., 2018) manages to create a lower-dimensional space by mapping high-dimensional data to lower dimensions through pairwise probability similarities while preserving both local and global structure. Gaussian distributions are used to compute the conditional probabilities that represent the similarities between the points in the original space. The t-distribution is used to compute the low-dimensional space's similarity probability distribution. T-SNE minimises the Kullback–Leibler divergence between input feature space and the reduced feature space by using the two distributions.

Spectral embedding (Belkin & Niyogi, 2003) constructs a weighted graph representing the data and uses its Laplacian matrix for dimensionality reduction to preserve local structures. The graph connects each point to its nearby points, computes the graph Laplacian matrix, and finds its eigenvectors corresponding to the smallest non-zero eigenvalues. These eigenvectors form the low-dimensional embedding.

Diffusion Map (Berry and Harlim, 2016) uses diffusion processes on the manifold to capture the intrinsic structure of the data. A graph is constructed, where edges represent the probability of transitioning between points in a random walk. The eigenvectors of the normalised graph Laplacian are computed, which correspond to different time scales of the diffusion process. These eigenvectors are the low-dimensional embeddings created by preserving diffusion distances.

PHATE (Moon et al., 2019) models diffusion processes through heat kernels to create an embedding that captures the intrinsic structure of the data. A neighbourhood graph is constructed, and local affinities are computed. Diffusion is applied to capture multi-scale relationships, potential distances that preserve both local and global structure are computed and embedded into low dimensions using MDS.

UMAP (McInnes et al., 2020) maps the high-dimensional data into low-dimensional embeddings that have a similar topological structure. A weighted graph is constructed with edges representing k-nearest neighbours. The edge weights are assigned using a fuzzy set membership function based on distance, thus creating a fuzzy topological representation. A low-dimensional space is initialised (usually using spectral techniques), and a similar graph is created (with different edge weights). Finally, UMAP optimises the low-dimensional space to minimise the cross-entropy between the two graphs. Recently, UMAP has been applied to spike sorting with promising results [47 48].

TriMap (26) creates embeddings based on triplet constraints, which compare relative proximities between points. TriMap samples triplets of points *(i, j, k),* where *i* should be closer to *j* than to *k* in the embedding space. It then optimises an objective function that minimises violations of these constraints using gradient descent. By focusing on these relative proximity relations rather than absolute distances, TriMap efficiently captures both local and global structure.

### Clustering Algorithms

External evaluation metrics require both the predicted cluster labels and the corresponding ground truth labels. As such, we employed K-Means (MacQueen, 1967) clustering to obtain the labels necessary for external metrics, applying it immediately after feature extraction. K-Means has long been used in clustering, with many adaptations developed over time. It was first utilized for spike sorting in 1988 (Salganicoff et al., 1988; Veerabhadrappa et al., 2020) and has remained the standard method for many years. Even recent spike sorting pipelines (Caro-Martín et al., 2018; Pachitariu et al., 2016) either rely on or are inspired by K-Means, and in a recent comparative study of 25 clustering methods, it demonstrated its continued competitive performance by ranking third (Veerabhadrappa et al., 2020).

K-Means (MacQueen, 1967) is a centroid-based clustering technique that achieves clustering by dividing the data space into *k* groups and allocating each point to the closest centroid according to Euclidean distance. However, it has a number of drawbacks. First of all, it necessitates pre-specifying the number of clusters, which can be difficult for real-world data. Nevertheless, there are preprocessing methods for finding the *k* parameter. Second, the method is non-deterministic in its most basic version, which means that different outcomes may be obtained from repeated executions. This problem has been resolved by more recent improvements that have increased its consistency. Thirdly, overlapping clusters are hard for K-Means to handle. However, this disadvantage is beneficial for our analysis: the more performant feature extraction methods will improve cluster separation, which will be shown by a higher K-Means performance.

### Performance Metrics

It is worth mentioning that, despite its application in evaluating spike sorting methods (Eom et al., 2021; Radmanesh et al., 2022), accuracy is not a suitable performance metric. The primary issue with accuracy is that spike sorting is an unsupervised task, where ground truth labels are not present. Since accuracy requires the ground truth labels to evaluate performance, it is impractical. Additionally, as previously stated, neuronal data is inherently unbalanced due to the different firing rates of neurons, and it has been widely demonstrated that accuracy is inadequate in measuring performance on imbalanced datasets (Joshi et al., 2001; Sun et al., 2009; Wegier & Ksieniewicz, 2020; Weiss, 2004). Nonetheless, the selected metrics allow us to assess cluster separation and structure using internal metrics and to evaluate the “accuracy” of clustering through external metrics.

Six metrics were used to evaluate the outcomes; the first three are external measures, while the latter three serve as internal measures (Rendón et al., 2011). They are: Adjusted Rand Index (ARI), Adjusted Mutual Information (AMI), V-Measure (VM), Calinski-Harabasz Score (CHS), Davies-Bouldin Score (DBS), and Silhouette Score (SS). Since spike sorting ends in clustering, these clustering metrics are suitable for evaluating the quality of feature extraction. External metrics measure how accurately the clustering algorithm recovers known class labels; they reflect how distinctly features separate the data. If features are perfectly separated, the clustering algorithms typically achieve high external scores. Internal metrics measure cluster compactness, separation, and shape as independent of ground truth (Ardelean et al., 2024); therefore, they are appropriate for judging feature extraction when synthetic datasets provide true labels. Internal metrics describe cluster structure, while external metrics measure agreement with true labels.

Using multiple measures instead of one index helps us consider different aspects of clustering performance. Table 1 gives the intuitive meaning for each metric and its scoring range. A technique that does well across these varied measures suggests a sounder and more even approach to feature extraction, lessening the chance of bias in the assessment.

**Table 1:** A short description of each performance evaluation metric, specifying its type and range. It is noteworthy to mention that DBS has an inverse range where higher values represent a worse result

Name	Type	Description	Range [worst, best]
ARI	External	Chance‐corrected score based on pairwise comparisons of objects, rewarding when pairs are either consistently grouped together or separated in both predicted and ground‐truth clusters	[−1, 1]
AMI	External	Chance‐corrected score based on entropy‐based mutual information between the predicted and actual labels, with an adjustment for the expected value under random assignments	[0, 1]
Purity	External	Fraction of correctly assigned points across all clusters by assigning each cluster to the majority true class within it	[0, 1]
DBS	Internal	Average similarity ratio of each cluster with its most similar cluster, where similarity is defined as the sum of within‐cluster scatter relative to between‐cluster separation	(Inf, 0]
CHS	Internal	Ratio of between‐cluster dispersion to within‐cluster dispersion, normalized by the number of clusters and total points	[0, Inf)
SS	Internal	Average across all data points for the normalized difference between its mean intra‐cluster distance and lowest mean inter‐cluster distance	[−1, 1]

#### External Metrics

External metrics require the ground truth labels to be compared with the predicted labels. Furthermore, all these metrics are bounded with higher values being more desirable.

ARI (Hubert & Arabie, 1985; Steinley, 2004; Vinh et al., 2009) extends the Rand Index (RI) metric to account for chance agreements. Essentially, RI (Fowlkes & Mallows, 1983) computes is score as a pairwise comparison whether both set of labels (predicted and true) are aligned (agreements where both consider two data points in the same cluster or in different clusters) or not (disagreements). The following formulas describe the computation of these metrics:

1 $$RI= \frac{agreements}{agreements+disagreements}$$

2 $$ARI= \frac{RI-ExpectedRI}{MaxRI-ExpectedRI}$$

Here, *ExpectedRI* is the expected score if clusters were assigned randomly, estimated via a contingency table using permutations, *MaxRI* is 1, the maximum value of the score (Hubert & Arabie, 1985).

AMI (Strehl & Ghosh 2002; Vinh et al., 2009) extends the Mutual Information (MI) (Steinmetz et al., 2021) metric by incorporating entropy (*H*) into its computation. AMI also incorporates the normalization component (Lazarenko & Bonald, 2021; Vinh & Epps, 2010; Vinh et al., 2009) of Normalized Mutual Information. It measures the mutual dependence between two clusters and is described by the following equations:

3 $$MI\left(U, V\right)=\sum\limits_{i=0}^{\left|U\right|}\sum\limits_{j=0}^{\left|V\right|}\frac{\left|Ui\cap Vj\right|}{N}loglog \frac{N\left|Ui\cap Vj\right|}{\left|Ui\right|\left|Vj\right|}$$

4 $$AMI= \frac{MI\left(U, V\right)-E\left(MI\left(U,V\right)\right)}{average\left(H\left(U\right),H\left(V\right)\right)-E\left(MI\left(U,V\right)\right)}$$

Here, *U* and *V* are the two clusters, *N* is the total number of data points and *|X|* is the size of a given subset *X*.

Purity (Manning et al., 2008; Rendón et al., 2011) computes the percentage of samples clustered correctly. This is computed as the ratio between the sum of the maximum intersections between the true and predicted labels for each cluster by the total number of samples. Thus, Purity can be viewed as a measure of how many of the samples of the predicted cluster belong to a single true cluster. The following formulas describe the computation of this metric:

5 $$Purity=\frac{1}{N}\sum\limits_{i=1}^{k}max\left|{C}_{i}\cap L\right|$$

Here, *N* represents the total number of samples in the dataset, *k* is the number of clusters in the set of predicted labels, *C*_*i*_ represents the samples of a cluster, *i*, of the predicted set of labels and *L* is the set of true labels.

#### Internal Metrics

Internal metrics do not require a ground truth to be available. They evaluate the intra-cluster and inter-cluster distances, thus evaluating the morphology of the clusters. Thus, internal metrics are biased toward dense and well-separated clusters. Even correct clusterings in which clusters do not respect these criteria can receive lower scores. Internal metrics were used with the ground truth labels to evaluate the synthetic datasets. This results in an evaluation of the capabilities of feature extraction methods to generate clusters (based on the true labels) that are dense and well-separated.

DBS (Caliński & JA H., 1974; Davies & Bouldin, 1979; Halkidi et al., 2001) is computed as the average similarity of clusters. The similarity is computed using the distance between clusters and their sizes. DBS has an inverse performance interval to the other metrics presented in this work. It has only a lower bound at 0, and lower values represent a higher performance. The following formulas describe the computation of this metric:

6 $${R}_{i,j}=\frac{{s}_{i}-{s}_{j}}{{d}_{i,j}}$$

7 $$DBS= \frac{1}{k}\sum\limits_{i=1}^{k}max{\mathrm{R}}_{i,j}$$

Here, *R* represents the similarity between clusters *i* and *j*, *s*_*i*_ is the mean of all distances between the points of cluster *i* and its centroid, *d*_*i,j*_ is the distance between clusters *i* and *j* given by their centroids, and *max(R*_*i,j*_*)* is the maximum similarity of clusters *i* and *j*.

CHS (Rendón et al., 2011; Rosenberg, 2007), or Variance Ratio Criterion, is computed as the ratio between the intra-cluster to inter-cluster dispersion. The dispersion is based on the sum of squared distances. For this metric, a higher value indicates a better result and it has no upper bound. The following formula describes the computation of this metric:

8 $$CHS=\frac{tr\left(Bk\right)}{tr\left(Wk\right)}*\frac{n-k}{k-1}$$

Here, *tr(X)* is the trace of the dispersion matrix (either between *Bk* or within *Wk*), *n* is the dataset size and *k* is the number of clusters.

SS (Rosenberg, 2007; Rousseeuw, 1987) is computed as the ratio between the mean distance between a point and the rest of the points of that cluster and the mean distance between the point and all the points of the nearest cluster. SS has an interval of [−1, 1] where 1 represents well-separated dense clusters, 0 overlapping clusters, and −1 an incorrect clustering. Thus, SS evaluates as correct (and outputs higher scores for) the traditional structure of clusters. The following formula describes the computation of this metric:

9 $$SS= \frac{b-a}{\mathrm{max}\left(a,b\right)}$$

Here, *b* is the mean of all distances between a point in cluster *i* and all points of the closest cluster *j*, and *a* is the mean of all distances between a point in cluster *i* and all other points in the same cluster.

#### Synthetic Data

Ninety-five synthetic datasets (Pedreira et al., 2012), referred to as simulations (by the authors), were used in the analyses presented in this work. These datasets (Pedreira et al., 2012) were created by the Department of Engineering, University of Leicester, UK and are publicly available. Each dataset is derived from 594 distinct spike waveforms obtained from real brain recordings of a monkey. The original publication (Pachitariu et al., 2016) also investigated the ability of various clustering algorithms on these datasets, and it was found that, at best, they were able to identify 10 out of 20 true units. Details about the number of ground truth clusters and spikes of each simulation can be found in Table S2 in the Supplementary Material section. In average, the synthetic datasets contain 10–11 clusters with ~ 9300 spikes.

Initially, the spike waveforms comprised 316 samples at a 96 kHz sampling frequency. However, the datasets were downsampled to 24 kHz, yielding 79 samples per spike. Each of these datasets consists of a varying number of 2 to 20 single unit clusters and a multi-unit cluster. The single-unit clusters lie within 0 and 50 μm of the electrode, their amplitudes are normally distributed and scaled between 0.9 and 2 to mimic real data, and their firing rate follows a Poisson distribution with a mean between 0.1 and 2 Hz. The multi-unit cluster introduces complexity into the dataset. It was created through the aggregation of 20 unique neurons (thus, different spike shapes) within 50-140 μm of the electrode, their amplitude was fixed to 0.5 with a collective firing rate of 5 Hz (while each unique neuron fires at 0.25 Hz under an independent Poisson process). Spikes never overlap in time, and it was ensured that spikes have a time separation of at least 0.3 ms. Each individual cluster count has 5 independent datasets, meaning that there are 5 datasets with 2 single unit clusters, 5 with 3 single unit clusters and so on. Each of these synthetic datasets carries with it a set of ground truth labels. This allows for the evaluation of performance using external metrics as well.

**Fig. 1:**
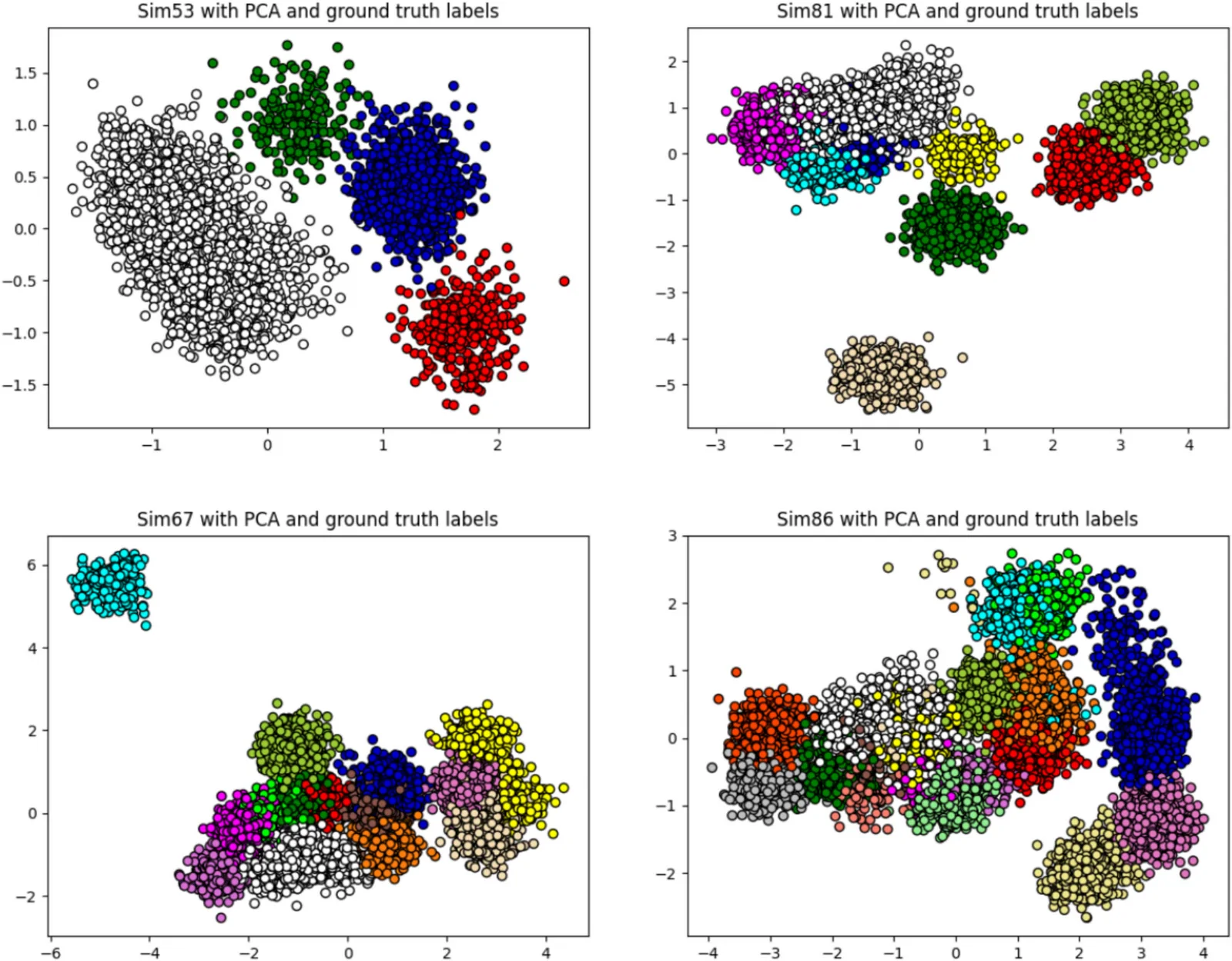
Synthetic datasets are presented with PCA and ground truth labels. Four different simulations were reduced to a 2-dimensional space using PCA. The colors represent the true clusters indicating that PCA is unable to find a set of features that offer cluster separability

A detailed comparison of the methods was made. Four representative simulations have been chosen for their variety in cluster count from the 95 datasets, allowing for the performance evaluation of feature extraction methods covering a wide range. The selected set of simulations can be viewed in Fig. 1, PCA was used to obtain a 2-dimensional representation. A short description of each of these simulations follows:

- Simulation 53 (Sim53—Fig. 1) is composed of 4490 spikes distributed in 3 single-unit clusters and a multi-unit cluster (a total of 4). This dataset was used to visualise the impact of alignment on feature extraction.
- Simulation 81 (Sim81—Fig. 1) is composed of 7937 spikes distributed in 8 single-unit clusters and a multi-unit cluster (in total 9).
- Simulation 67 (Sim67—Fig. 1) is composed of 11,377 spikes distributed in 13 single-unit clusters and a multi-unit cluster (in total 14).
- Simulation 86 (Sim86—Fig. 1) is composed of 13,847 spikes distributed in 18 single-unit clusters and a multi-unit cluster (in total 19).

A general comparison of the methods was also made, where all 95 datasets were analysed.

#### Data Preprocessing

The spikes obtained from these datasets have gone through a preprocessing step before applying the feature extraction methods. Alignment of spikes to their amplitude can help in the separation of clusters by feature extraction methods. The result of this process is presented in Fig S1 where the effect on the spikes and on the PCA transformation of the spikes can be viewed. All spikes have been shifted such that the amplitude, or maximum peak, can be found at a given index. This formula allows for the alignment of any point of reference, such as the minimum peak (Dipalo et al., 2017), to any chosen position.

### Real Data

The spe‑1 dataset (Marques-Smith et al., 2018, 2020) provides a rare ground‑truth resource by recording from the same cortical neuron in rats anesthetized with urethane using simultaneous patch‑clamp and high‑density 384‑channel CMOS extracellular probes. Across primary motor and somatosensory cortex, 43 neurons were targeted out of which 38 were recorded in cell‑attached mode and 5 in whole‑cell, yielding clear extracellular action potentials for 21 neurons—10 of which exhibited peak‑to‑peak amplitudes over 50 µV—thereby enabling direct validation of spike‑sorting algorithms. For each neuron, the dataset includes high‑pass–filtered (300 Hz) extracellular voltage traces alongside intracellular patch‑clamp recordings.

Two datasets were chosen from the 43 available, specifically c28 and c37. The raw recordings were band-pass filtered in the 300–7000 Hz range, and the spikes were extracted using the traditional amplitude thresholding of the standard deviation of the filtered signal multiplied by a factor of 4.

## Results

### Performance Evaluation on all Synthetic Datasets

**Fig. 2:**
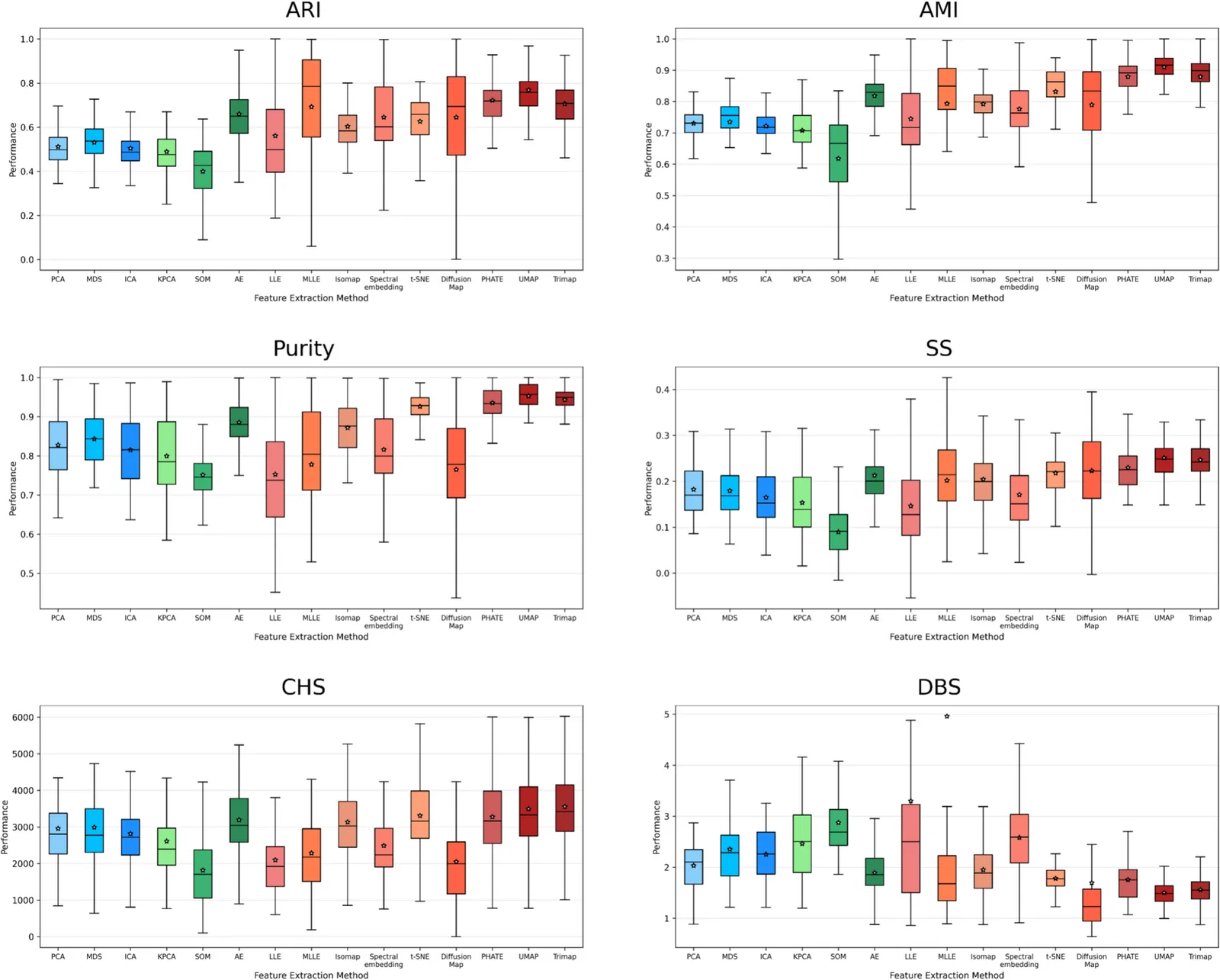
Performance evaluation of all feature extraction methods for all 95 datasets (star represents mean value, middle line represents the median value)

Due to the variability of the datasets, aggregating the results of all datasets (Pedreira et al., 2012) allows for a comprehensive evaluation of the feature extraction methods. In Table 2, using the Borda rank aggregation (Dwork et al., 2001), the feature extraction methods have been ranked based on the performance obtained across all datasets for each metric. In Fig. 2, the scores obtained by each algorithm for the 6 performance metrics are presented in box plots. Both of these analyses confirm the previous observations made for the selected array of datasets. From the perspective of both the external and internal metrics (DBS has an inverse range, and lower values are better), UMAP is the highest-performing feature extraction method, followed closely by TriMap and PHATE. This is confirmed by both the high scores obtained for each metric and the low variance they present across the 95 datasets (as shown by Fig. 2). AE and t-SNE scores are slightly lower, yet still significantly higher than all the other methods.

**Table 2:** Borda ranking by each performance metric across all 95 datasets

Method	ARI	AMI	Purity	SS	CHS	DBS
1	UMAP	UMAP	UMAP	Trimap	Trimap	UMAP
2	PHATE	Trimap	Trimap	UMAP	UMAP	Diffusion Map
3	Trimap	PHATE	PHATE	PHATE	t-SNE	Trimap
4	MLLE	t-SNE	t-SNE	t-SNE	PHATE	PHATE
5	AE	MLLE	Isomap	Diffusion Map	Isomap	t-SNE
6	Diffusion Map	AE	AE	MLLE	AE	MLLE
7	t-SNE	Diffusion Map	MDS	AE	PCA	AE
8	Spectral embedding	Isomap	PCA	Isomap	MDS	Isomap
9	Isomap	Spectral embedding	Spectral embedding	PCA	ICA	PCA
10	MDS	MDS	MLLE	MDS	KPCA	LLE
11	LLE	LLE	ICA	Spectral embedding	Spectral embedding	MDS
12	PCA	PCA	Diffusion Map	ICA	MLLE	ICA
13	ICA	ICA	KPCA	LLE	Diffusion Map	Spectral embedding
14	KPCA	KPCA	LLE	KPCA	LLE	KPCA
15	SOM	SOM	SOM	SOM	SOM	SOM

The linear and traditional non-linear (except AE) methods obtain scores that are generally lower with SOM having the worse performances and the highest variance across datasets. LLE, MLLE and Diffusion Map may obtain high scores for some datasets, their variance on the 95 datasets indicates that they are not an adequate general approach for spike sorting.

**Fig. 3:**
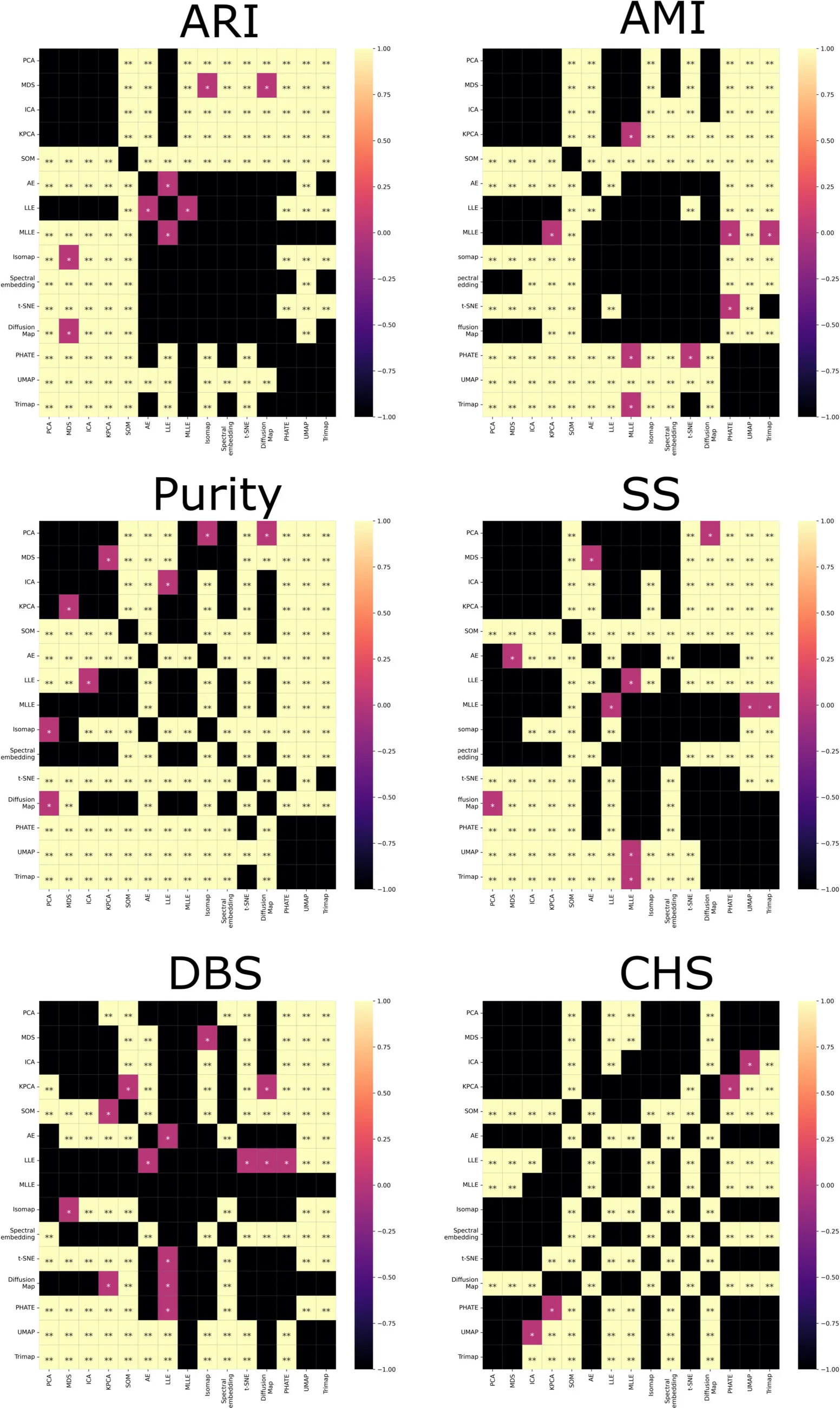
P value of t-tests (with a Bonferroni correction) for each of the metric on all 95 simulations (** represents p < 0.01, * represents 0.01 < p < 0.05, while no text represents 0.05 < p)

As further validation of the results obtained, a statistical analysis using t-tests with a Bonferroni correction was performed which can be viewed in Fig. 3. Our analysis indicates that there is no statistically significant difference between the 3 highest performing feature extraction methods, PHATE, UMAP and TriMap from the perspective of all metrics (except DBS which indicates that PHATE is significantly different). This analysis also indicates that PCA, MDS, ICA, KPCA, LLE, and MLLE have no statistically significant difference among them. As expected, the SOM algorithm is statistically different to all other methods, but its scores are rather disappointing. The AE method varies across metrics in its statistical difference, yet most commonly it appears to be similar to t-SNE and Isomap, which is confirmed by the previous analyses made.

The statistical comparisons in our study were conducted over a large sample size (n = 95 datasets per method comparison), which mitigates the impact of non-normal distributions due to the Central Limit Theorem as they are considered robust to moderate violations of the normality of samples. We used Welch’s t-test, which is specifically designed for cases where the assumption of equality of variance may be violated. Welch’s t-test adjusts the degrees of freedom based on the sample variances and sizes, providing a more reliable test under variance inequality. We also employed the non-parametric Mann–Whitney U test as an alternative to the independent samples t-test. The Mann–Whitney U test results can be visualized in Fig S2 in the Supplementary Material.

The analyses of this large array of synthetic datasets (Pedreira et al., 2012) is revealing towards identifying the most performant feature extraction method. However, being synthetic datasets, they do not include all complexities of real spike datasets. These datasets were generated with an integrated refractory period of at least 3 ms between any pair of spikes (Pedreira et al., 2012), this reduces the complexity of these datasets as no spike collisions or bursting activity occurs. However, as stated by the creators of these datasets (Pedreira et al., 2012) in their analyses, no clustering algorithm was capable of identifying of more than 10 clusters out of the maximum of 20. Despite the fact these datasets do not include all the complexities of real data, it is clear that they contain enough complexity to be a feasible choice for analysis.

### Performance Evaluation of Individual Synthetic Datasets

The analysis starts with the four selected datasets (Pedreira et al., 2012). All feature extraction algorithms have been run on each of these four datasets. This analysis allows for the assessment of the performance of the feature extraction methods for varying numbers of clusters. The parametrization of each algorithm used in the analyses can be found in Table S1 in the Supplementary Material.

**Fig. 4:**
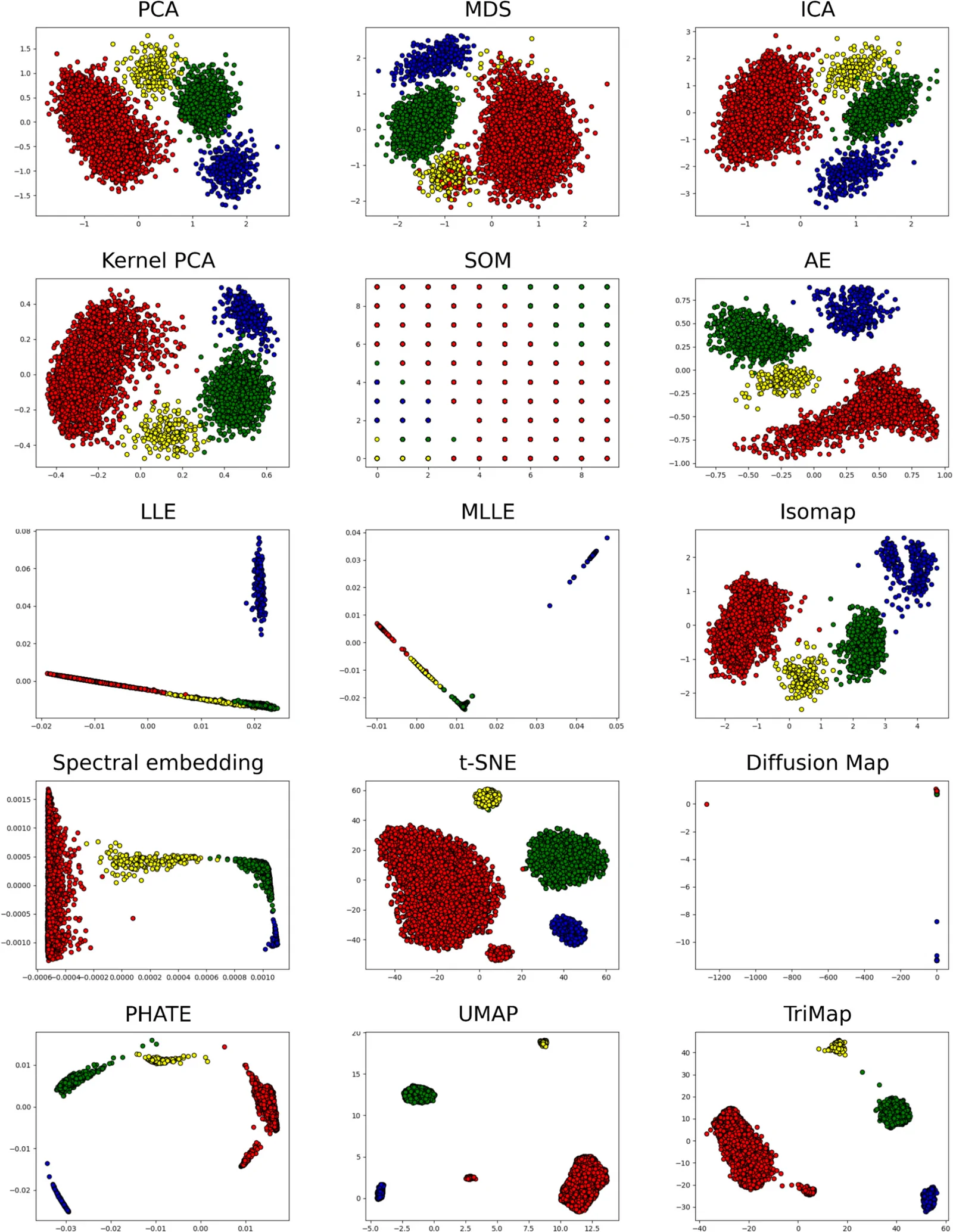
Feature extraction methods applied on the Sim53 dataset. Colors represent the ground truth labels such that the amount of separability between clusters offered by each individual feature extraction method is easily visible

The analysis of the feature extraction methods on the Sim53 dataset, which contains only 4 clusters, indicates that linear feature extraction methods, although extensively used in spike sorting, cannot capture the inherent complexities of neural data. This can be seen particularly in the scores obtained from the perspective of external metrics in Table 3. However, by comparing the spaces obtained in Fig. 4, the clusters may be separable by another clustering algorithm to obtain better scores. Non-linear methods do not fare any better either. The SOM obtains the lowest scores across all metrics, while KPCA is on par with its linear version in both scores in Table 3 and by the visual inspection of Fig. 4. The non-linear space obtained by AE separates clusters, yet it distorts their shape.

**Table 3:** Comparison of feature extraction methods on Sim53 (containing 4 clusters) from the perspective of the six performance evaluation metrics

Algorithm	ARI	AMI	Purity	SS	CHS	DBS
PCA	0.52	0.701	0.953	0.205	**1635.47**	1.655
MDS	0.465	0.597	0.905	0.156	1430.06	2.052
ICA	0.521	0.705	0.951	0.199	1611.126	1.686
KPCA	0.518	0.69	0.949	0.201	1620.848	1.691
SOM	0.362	0.503	0.837	0.025	594.599	6.263
AE	0.736	0.79	0.957	0.21	1479.332	1.588
LLE	**0.999**	**0.998**	**1**	0.317	1496.737	1.155
MLLE	**0.999**	**0.995**	**0.999**	0.317	1497.1	1.158
Isomap	0.536	0.697	0.92	0.187	1463.637	1.687
Spectral embedding	0.534	0.714	0.957	0.212	1632.349	1.653
t-SNE	0.488	0.67	0.924	0.188	1372.51	2.039
Diffusion Map	0.959	0.931	0.957	**0.338**	1260.056	**0.981**
PHATE	0.531	0.735	0.957	0.202	1613.265	1.659
UMAP	0.911	0.901	0.968	0.311	1383.718	1.435
Trimap	0.508	0.716	0.929	0.203	1517.197	1.578

Regarding non-linear manifold methods, LLE and MLLE are the best performing algorithms from the perspective of external metrics. Yet, when visually inspecting the spaces provided, there is no true separation of the clusters. This indicates that this is an exception in which even without true separation the K-Means clustering algorithm was able to partition the dataset correctly into clusters. However, another clustering algorithm such as DBSCAN will not be able to obtain such high scores on such a feature space. Diffusion Map obtains the best results when considering the SS and DBS metric, indicating that it creates the most dense and well-separated clusters. However, by comparing the results presented in Table 3 and Fig. 4, it is clear that Diffusion Map tends to create a feature space where the points are extremely dense (most points are duplicated). The internal metrics correctly evaluate that this would indeed be an easily separable space, and this is confirmed by the external metrics, which show that the true clusters are actually correctly identified through the feature space created by the Diffusion Map. However, this may become problematic for more clusters as multiple clusters may be projected to the same point leading to the phenomenon of underclustering. Moreover, the embedding space obtained overlaps perfectly with the SS and DBS cluster concepts due to the low intra-cluster and high inter-cluster distances.

Another great competitor for this dataset is UMAP which obtains high scores for all the metrics, this is confirmed through visual inspection, and the clusters are consistent with the true labels and are dense. UMAP, Trimap, PHATE and t-SNE have the same tendency to split the true red cluster into two subclusters, this tendency may be due to the different spike shapes found in the multi-unit red cluster.

The other non-linear manifold approaches perform similarly to linear approaches. Isomap separates the clusters similarly to linear approaches, yet it segments the blue cluster, while Spectral embedding seems to cut off the embedding space on the multi-unit red cluster.

**Fig. 5:**
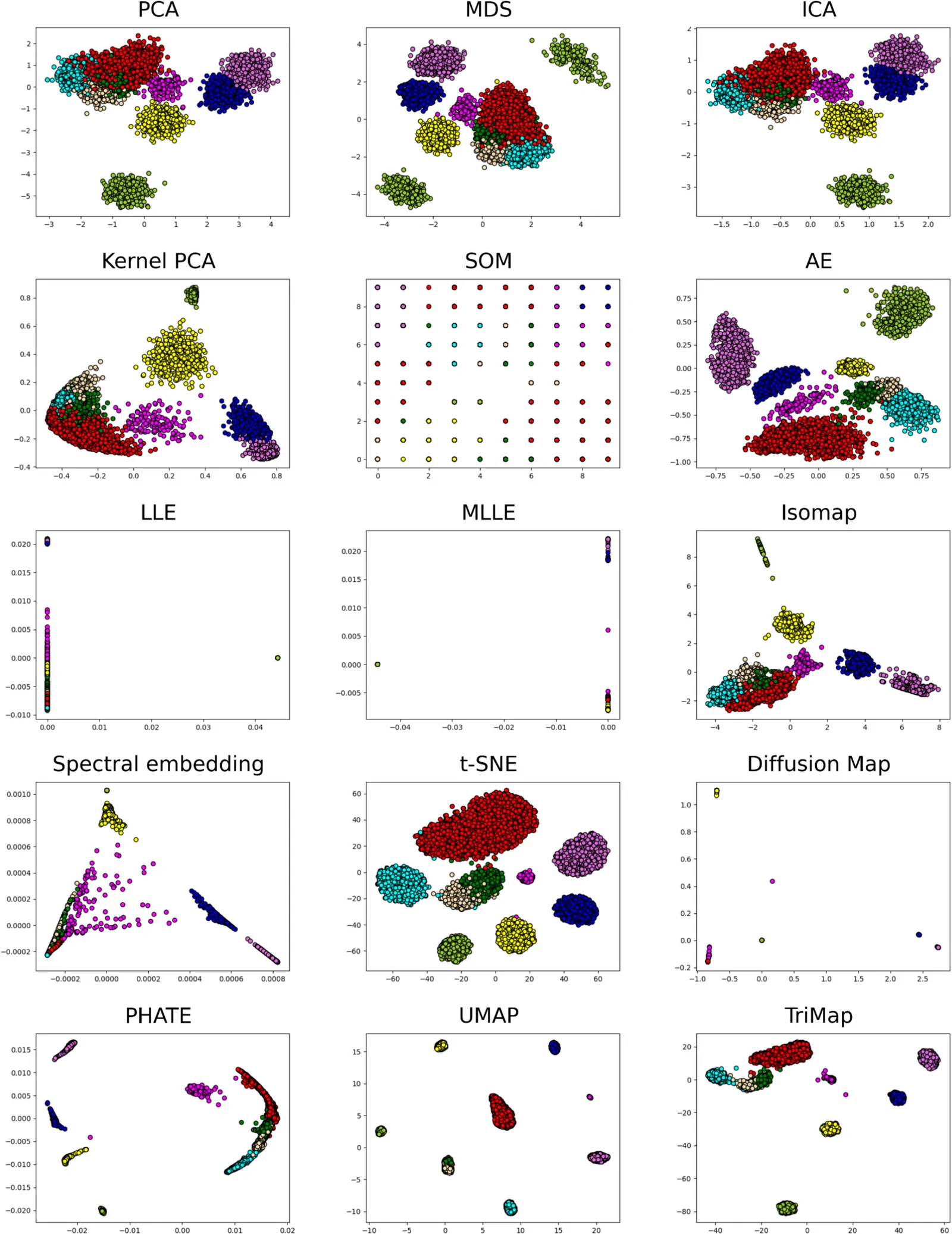
Feature extraction methods applied on the Sim81 dataset. Colors represent the ground truth labels such that the amount of separability between clusters offered by each individual feature extraction method is easily visible

Table 4 shows a similar trend to the previous dataset when analysing the performance of the feature extraction methods on Sim81, which contains 9 clusters, where Diffusion Map and UMAP have very high performance. By visual inspection in Fig. 5, it can be seen that Diffusion Map creates extremely dense clusters (most embeddings are duplicated), while UMAP creates well-separated dense clusters that are closer to the traditional concept of a cluster.

**Table 4:** Comparison of feature extraction methods on Sim81 (containing 9 clusters) from the perspective of the six performance evaluation metrics

Algorithm	ARI	AMI	Purity	SS	CHS	DBS
PCA	0.528	0.744	0.795	0.23	5407.828	1.841
MDS	0.619	0.774	0.813	0.207	5327.273	6.976
ICA	0.462	0.711	0.804	0.197	5317.578	2.099
KPCA	0.562	0.767	0.844	0.226	5310.696	1.946
SOM	0.481	0.697	0.83	0.193	3954.929	1.861
AE	0.731	0.886	0.926	0.287	5942.182	1.326
LLE	0.515	0.737	0.784	0.216	4384.025	2.421
MLLE	0.513	0.786	0.756	0.274	3752.723	**1.093**
Isomap	0.647	0.819	0.903	0.266	5759.539	1.423
Spectral embedding	0.609	0.789	0.806	0.255	4688.755	2.259
t-SNE	0.721	0.885	0.948	0.28	5822.569	1.431
Diffusion Map	**0.802**	0.88	**0.867**	**0.294**	4973.175	1.205
PHATE	0.725	**0.9**	0.933	0.287	6008.121	1.314
UMAP	0.739	0.912	0.938	0.287	5996.497	1.3
Trimap	0.733	0.909	0.937	0.289	**6024.442**	1.298

However, some algorithms diverge in their performance with the increased complexity of a higher number of clusters. LLE and MLLE are some of the lowest-performing algorithms for the Sim81 dataset. This is expected when visualising the embedding spaces provided by these methods in Fig. 5, it seems that most embeddings have one of the features mostly unused for discrimination (x-axis). Moreover, t-SNE, PHATE, and TriMap obtain similar results to those of UMAP, by visual inspection, exactly the same clusters (green and beige) overlap in all the embeddings spaces generated by these methods. Yet, the most dense and separated clusters are those obtained by UMAP. This indicates that with the increased complexity of a higher number of clusters, these more complex models are able to find the intrinsic relationships between data points in order to create an embedding that truly represents the high-dimensional data.

The linear feature extraction methods are unable to find embeddings that allow for the separation of clusters as indicates by the low scores across all metrics except CHS with MDS obtaining the highest result which are still considerably lower than those obtained by the non-linear manifold approaches. Regarding the non-linear approaches, the SOM algorithm continues to underperform, and KPCA has no significant improvement over its linear version, both when comparing the scores and the embeddings. The AE is able to create a separable space that is similar to those obtained by the best non-linear manifold approaches, yet the clusters are slightly more dispersed.

**Fig. 6:**
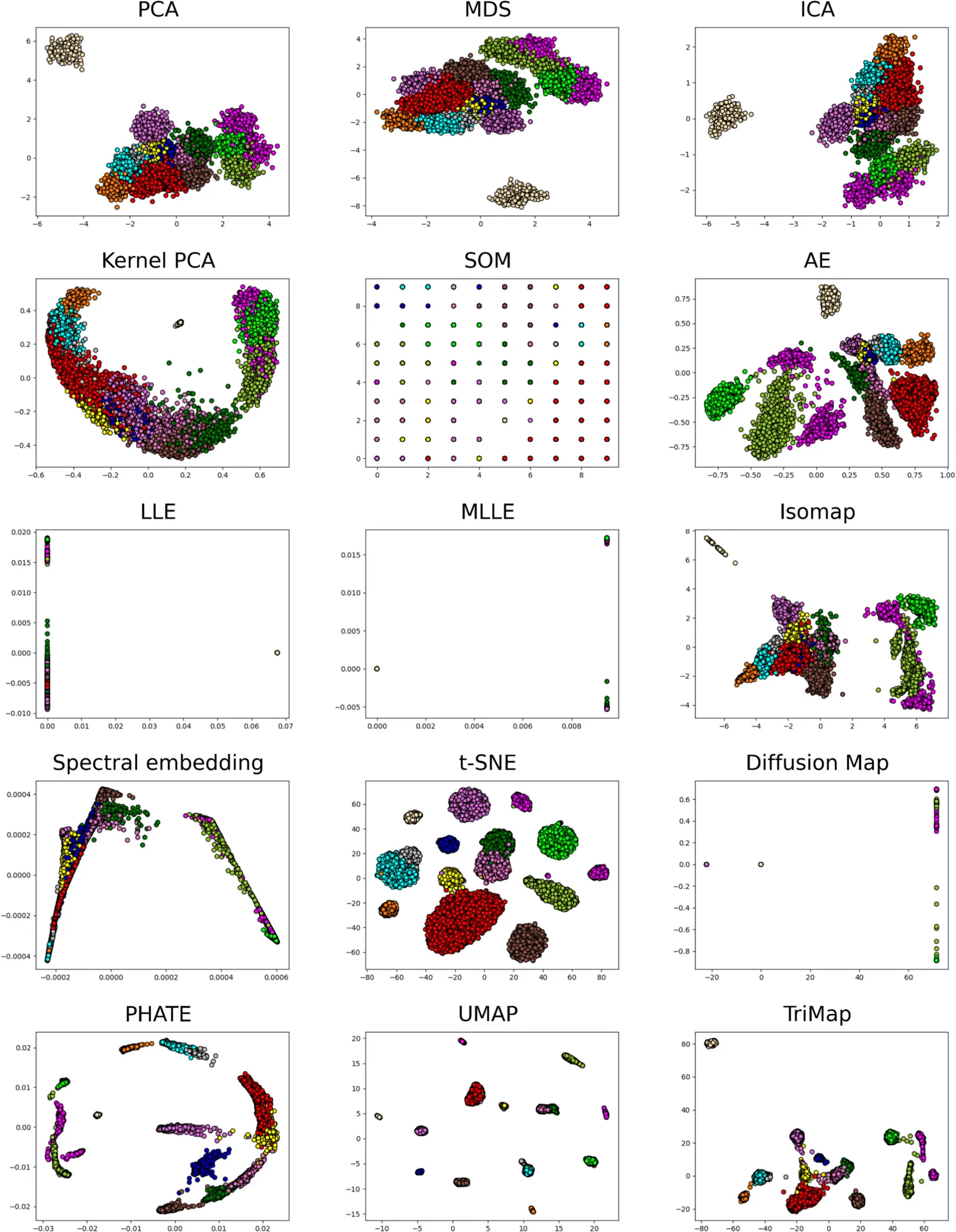
Feature extraction methods applied on the Sim67 dataset. Colors represent the ground truth labels such that the amount of separability between clusters offered by each individual feature extraction method is easily visible

Table 5 shows the results obtained by the feature extraction methods on the Sim67 datasets, which contain 14 clusters, while Fig. 6 allows for the visual inspection of the embedding spaces obtained. The linear approaches continue to have a low performance; however, they do not seem to be affected by the increase in complexity as much as the manifold LLE/MLLE approaches, which had a severe decrease in performance as the number of clusters increased. All of the linear feature extraction methods seem capable of clearly separating a single cluster (beige), while the rest remain overlapping.

**Table 5:** Comparison of feature extraction methods on Sim67 (containing 14 clusters) from the perspective of the six performance evaluation metrics

Algorithm	ARI	AMI	Purity	SS	CHS	DBS
PCA	0.485	0.72	0.779	0.169	3338.865	1.875
MDS	0.554	0.774	0.815	0.206	3701.141	2.314
ICA	0.519	0.727	0.802	0.174	3528.657	1.787
KPCA	0.371	0.622	0.666	0.088	2722.653	2.735
SOM	0.409	0.653	0.694	0.069	1617.331	2.874
AE	0.679	0.847	0.855	0.233	4395.376	1.65
LLE	0.47	0.701	0.742	0.121	3108.751	2.523
MLLE	0.664	0.79	0.731	0.219	2649.706	1.348
Isomap	0.57	0.783	0.816	0.22	4224.48	1.711
Spectral embedding	0.573	0.768	0.786	0.147	2571.183	2.382
t-SNE	0.678	0.88	0.893	0.243	4582.876	1.538
Diffusion Map	0.163	0.522	0.499	**0.352**	1267.865	1.542
PHATE	**0.757**	**0.9**	**0.925**	0.264	4610.405	1.437
UMAP	**0.758**	**0.916**	**0.92**	0.272	**4792.607**	**1.314**
Trimap	**0.759**	**0.916**	**0.92**	0.275	**4804.884**	**1.311**

Regarding the traditional non-linear approaches, SOM continues to be unable to find embeddings that reproduce the high-dimensional relationships in the case of this dataset, while KPCA performs worse than PCA on this specific data from both the perspective of the scores and the embedding space. The AE is again able to create a mostly separable space, and it is comparable to t-SNE, yet still worse than the best performing non-linear manifold approaches.

A trend appears to emerge regarding the non-linear manifold approaches. This increase in complexity seems to favour the more complex models, specifically PHATE, UMAP, and TriMap, which manage to obtain the highest scores for all metrics except SS. MLLE and t-SNE have also seen an increase in performance, yet remain significantly lower in performance when compared to the previously mentioned algorithms. Conversely, Diffusion Map is unable to identify a separable space as indicated by the low performance of external metrics; however, it obtains the highest score for the SS metric due to the highly dense clusters generated. The issue that was aforementioned with the dense clusters generated by Diffusion Map becomes apparent in this case. Its tendency to project many spikes to the same low-dimensional point has in this case mapped multiple ground truth clusters to the same location which led to underclustering, an erroneous result. The other non-linear manifold approaches have comparable performance to linear approaches.

**Fig. 7:**
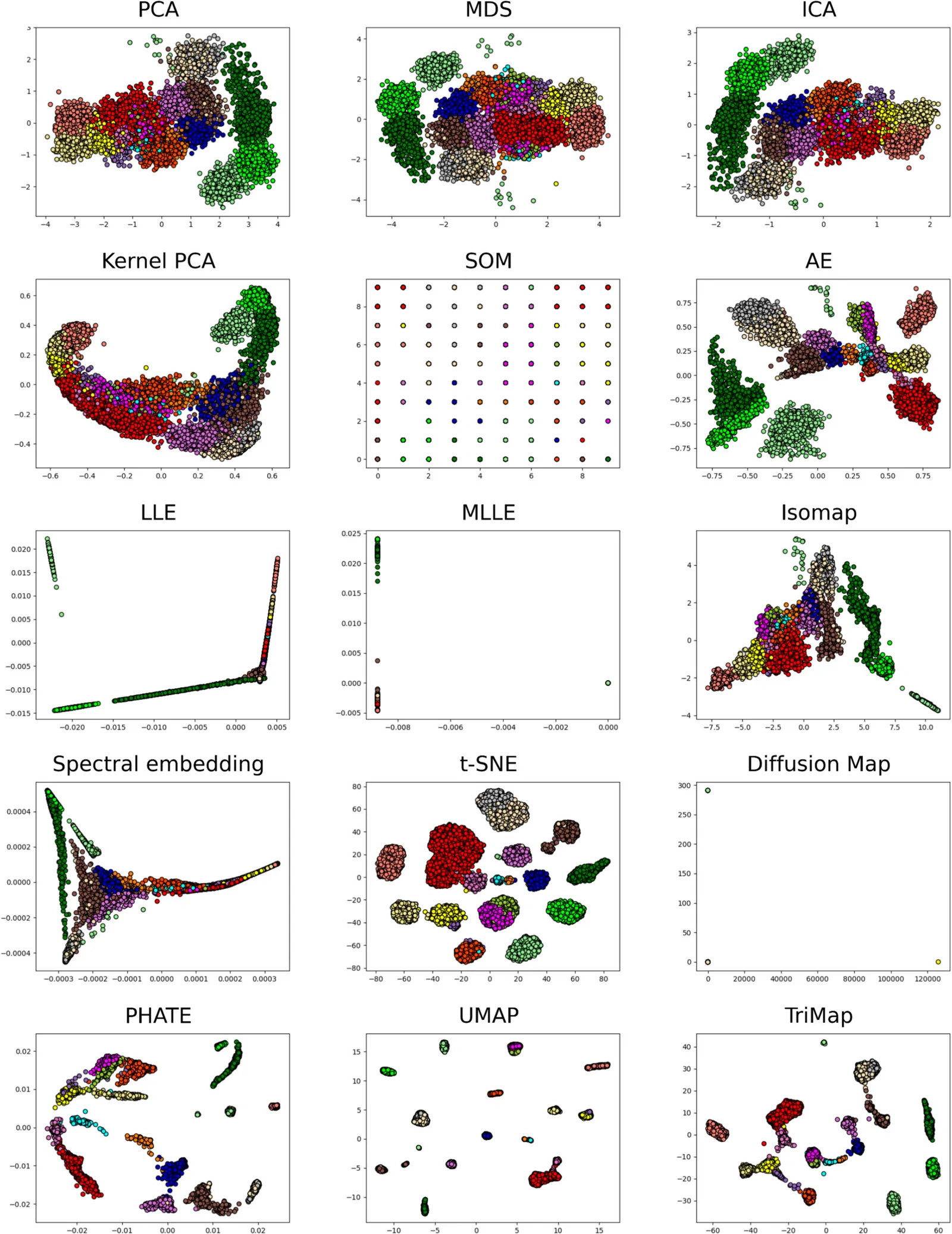
Feature extraction methods applied on the Sim86 dataset. Colors represent the ground truth labels such that the amount of separability between clusters offered by each individual feature extraction method is easily visible

The results of the analyses on Sim67 and Sim86 from Table 5 and Table 6 respectively, confirm that, although LLE, MLLE, Diffusion Map obtain high scores on datasets with a low number of clusters, as the number of clusters increases their performance degrades. Diffusion Map manages to obtain the highest scores for the SS and DBS metric. By comparing the results obtained in Table 6 and the space created in Fig. 7, the embeddings space obtained by these algorithms offer almost no separable clusters as they tend to be lines or pinpoints.

**Table 6:** Comparison of feature extraction methods on Sim86 (containing 19 clusters) from the perspective of the six performance evaluation metrics

Algorithm	ARI	AMI	Purity	SS	CHS	DBS
PCA	0.466	0.713	0.739	0.125	2859.273	2.733
MDS	0.583	0.783	0.832	0.144	3378.853	2.623
ICA	0.431	0.693	0.716	0.109	2760.434	2.939
KPCA	0.467	0.705	0.727	0.105	2821.59	3.132
SOM	0.483	0.74	0.729	0.091	2546.982	2.325
AE	0.658	0.839	0.846	0.16	3777.1	2.378
LLE	0.469	0.746	0.732	0.115	2760.308	2.822
MLLE	0.602	0.826	0.714	0.186	2323.771	1.891
Isomap	0.588	0.788	0.793	0.166	3518.915	2.597
Spectral embedding	0.5	0.718	0.694	0.11	2622.293	2.804
t-SNE	**0.781**	0.914	**0.954**	0.214	4286.09	1.871
Diffusion Map	0.317	0.652	0.475	**0.286**	1434.835	**0.897**
PHATE	0.707	0.907	0.905	0.25	4089.876	1.632
UMAP	**0.781**	**0.921**	0.939	0.24	**4461.659**	1.567
Trimap	0.683	0.902	0.902	0.254	4429.627	1.603

The more complex algorithms such as t-SNE, PHATE, UMAP and TriMap thrive when the complexity is increased indicated by the high scores obtained. This is confirmed through the visual inspection shown in Fig. 7, where the clusters are dense and spherically shaped. Isomap and Spectral embedding have scores similar to those of linear and traditional non-linear approaches and the embedding spaces are not much more separable.

Only AE from the non-linear group of methods is able to attain a similar performance to that of non-linear manifold approaches. Linear methods seem to be able to separate all the clusters into the same two groups for this dataset.

### Performance Evaluation on Real Datasets

As the real datasets contain a ground truth through the dual recording of extracellular activity and intracellular activity, in this analysis we will evaluate the ability of feature extraction algorithm to generate spaces where the known activity is separated from the rest of the activity. For this analysis, the result of K-Means clustering is not required as the internal metrics allow us to evaluate the separability based on the generated feature space and the labels of the known activity. However, we will present the result of K-Means clustering for visual inspection in figures. The *k* value of K-Means was chosen to obtain a better separation in which as many intracellular spikes as possible are separated into a single cluster.

**Fig. 8:**
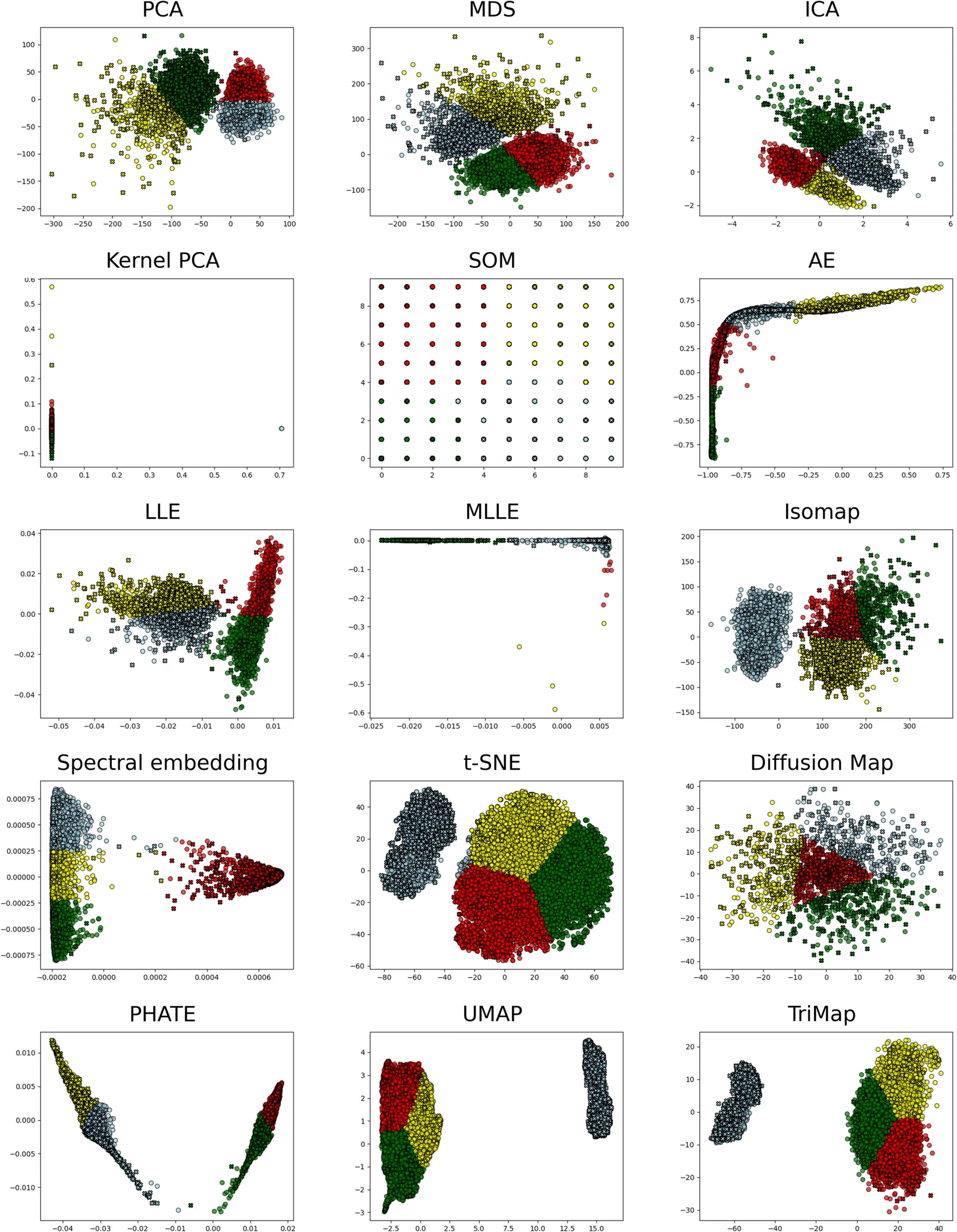
Feature extraction methods on the c28 real dataset. Colors represent the clustering labels and the ‘X’ maker represent the intracellular ground truth activity such that the amount of separability offered is easily observable

**Fig. 9:**
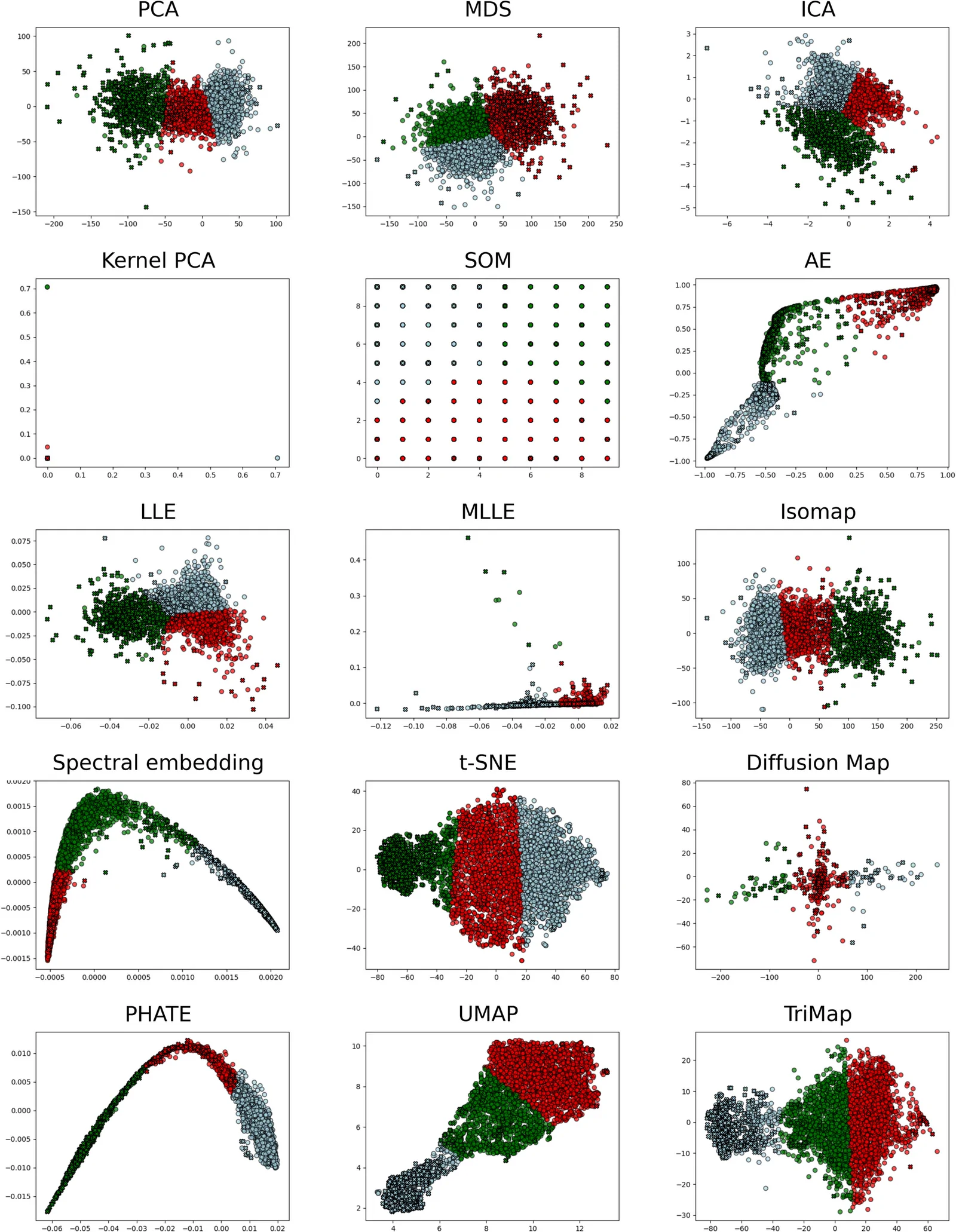
Feature extraction methods on the 37 real dataset. Colors represent the clustering labels and the ‘X’ maker represent the intracellular ground truth activity such that the amount of separability offered is easily observable

The scores obtained in the performance analysis of the two real datasets, c28 and c37, are presented in Table 7 and Table 8, respectively. UMAP, PHATE and TriMap continue to obtain the best results outperforming all other methods. However, for both datasets MLLE obtain the best score for the SS metric. Through the visual inspection offered by Fig. 8 and Fig. 9, it is clear to see that this happens due to the very dense space created which actually does not offer separation for the intracellular activity (denoted by the ‘X’ marker). Both Fig. 8 and Fig. 9 indicate that no method perfectly separates the intracellular ground truth activity (marked with ‘X’) as a single cluster as the points are spread over the whole space. However, through visual inspection, t-SNE, UMAP and TriMap are the methods that create spaces with the most conventional cluster shapes and the most separability for the intracellular activity. The linear approaches do not manage to create spaces that offer separability of the data as they tend to generate a single cluster. The traditional non-linear approaches create clusters of arbitrary shapes in the generated feature space which do not offer separability. For the real datasets, we have also included the execution time as the average of 5 runs for each algorithm.

**Table 7:** Performance analysis of feature extraction methods on the c28 real dataset. Bold values represent the highest score

Algorithm	SS	CHS	DBS	Time (s)
PCA	0.507	4220.086	0.85	0.003
MDS	0.415	3055.606	1.055	4142.04
ICA	0.423	2558.114	1.182	0.02
KPCA	0.281	9.001	5.65	0.98
SOM	0.162	1966.955	0.967	0.41
AE	0.074	864.956	1.672	25.86
LLE	0.37	2489.889	1.019	38.78
MLLE	**0.68**	3204.893	0.393	42.11
Isomap	0.547	5240.248	0.721	40.16
Spectral embedding	0.368	2975.995	0.793	63.15
t-SNE	0.327	3389.943	0.865	14.27
Diffusion Map	0.508	4.191	23.006	13.68
PHATE	0.666	8508.091	0.42	8.28
UMAP	0.625	**9128.739**	**0.412**	9.59
Trimap	0.569	7046.143	0.488	1.72

**Table 8:** Performance analysis of feature extraction methods on the c37 real dataset. Bold values represent the highest score of each metric

Algorithm	SS	CHS	DBS	Time (s)
PCA	0.597	4186.969	0.635	0.001
MDS	0.491	2360.420	0.808	499.62
ICA	0.485	1784.769	0.992	0.01
KPCA	−0.701	0.358	2.416	0.19
SOM	0.06	136.348	3.485	0.16
AE	−0.077	10.124	10.5	11.05
LLE	0.479	1767.493	0.907	4.39
MLLE	**0.741**	1914.077	0.512	5.40
Isomap	0.611	4670.308	0.592	5.61
Spectral embedding	0.515	2253.805	0.745	4.03
t-SNE	0.392	2124.513	0.699	4.69
Diffusion Map	0.500	0.944	15.929	5.15
PHATE	0.695	**6925.787**	**0.465**	5.15
UMAP	0.518	3483.85	0.55	11.72
Trimap	0.59	4480.079	0.48	0.78

## Conclusions

Our extensive analyses were made on a diverse array of feature extraction algorithms on a large number of synthetic (Magland et al., 2020).

datasets and real datasets from the perspective of 6 different performance evaluation metrics indicate that the non-linear manifold feature extraction approaches clearly outperform linear and non-linear approaches. Specifically, UMAP, TriMap, PHATE, and t-SNE are the most suitable approaches for spike sorting, with UMAP having the highest overall performance from both the scores obtained and through visual inspection. This statement is supported by our analysis of singular datasets, our global analysis of the 95 datasets, the rankings obtained, the statistical validation and the performance evaluation on real datasets. While other non-linear manifold approaches, such as Spectral embedding, Isomap and MLLE, outperform traditional linear and non-linear approaches, they still lag behind in performance. Spectral embedding is able to create a more interpretable space, yet the clusters are rarely separated.

LLE, MLLE, and Diffusion Map have shown good performance only on datasets with a low number of clusters, which renders them inadequate due to the increasing complexity of neuronal data given by recent developments in recording hardware. Moreover, through visual inspection, we can confidently say that the embedding spaces obtained are not interpretable, as there is almost no separation and no actual clusters can be seen. The high results obtained could be attributed to the linear separation offered by K-Means.

All the analyses performed indicate that traditional linear and non-linear approaches are inadequate for handling neuronal data. Specifically, SOMs are unable to integrate the high-dimensional relations between data points in their grid as their performance is low for all metrics. Although Kernel PCA integrates non-linearity, it does not seem to obtain a better performance than its linear counterpart. However, the AE manages to obtain results similar to those of t-SNE and Isomap offering separability which is confirmed by both the scores obtained and the visual inspection. Although mentioned in the ‘Materials and Methods’ section, we have chosen to leave out the HLLE, LTSA and non-metric MDS as their results were close to 0, indicating that these methods are not suitable for neuronal data.

The time analysis on real datasets indicates that although the non-linear manifold methods have an increased complexity, TriMap has the lowest execution time of about 1 s when considering performance but still higher than that of PCA of about 0.001 s. The other two highly performant methods, PHATE and UMAP, have a considerably higher execution time of about 5 s and 10 s, respectively which are still significantly lower than that of AE, LLE, MLLE and t-SNE which can reach execution times of > 30 s or MDS with execution times of > 100 s.

Our results indicate that TriMap offer the most robust and scalable feature extraction for spike sorting. For neuroscientists working with high-density probes or large-scale recordings, TriMap, UMAP or PHATE may be the most fitting options for feature extraction approaches due to their high performance and low execution time. For small datasets, methods like Diffusion Maps, LLE or MLLE may suffice. However, these tend to degrade with noise and increasing cluster count. Thus, for noisy or complex datasets with overlapping spikes or electrode drift, TriMap, UMAP and PHATE provide high separability, even under challenging conditions.

Evidently, other clustering algorithms may be used and may even obtain better results. However, we have chosen to analyze feature extraction algorithms and the separability of the embedding spaces that they offer. As such, a clustering algorithm such as K-Means, which separates any spaces in a linear manner, is a perfect candidate to explore the separability of any feature extraction method. Moreover, K-Means is one of the fastest algorithms and is commonly used in spike sorting, including some of the more recently developed spike sorters (Litke et al., 2004; Manning et al., 2008). Nevertheless, an analysis incorporating other clustering methods may be a future research direction.

Another future avenue of research is the investigation of advanced manifold approaches such as hierarchical (Bhatia et al., 2012; Marcílio-Jr et al., 2025) or multi-view extensions (Busch et al., 2023; Rodosthenous et al., 2021) of manifold learning. Hierarchical extensions of manifold learning offer the ability to capture multi-scale structures (Bhatia et al., 2012) that may be able to better handle to complexities of neural data as they can vary in temporal and spatial scales. Multi-view extensions of manifold learning, such as T-PHATE (Busch et al., 2023), can handle multiple ‘views’ of the same underlying neural activity, potentially integrating information from multiple electrodes or feature spaces. T-PHATE (Busch et al., 2023) has demonstrated a high performance in neural signal denoising and has been used in identifying brain-state trajectories in fMRI data.

One limitation of our work is the analysis of single-channel data as they suffer from multiple shortcomings. Single channel data lacks spatial information increasing the difficulty of distinguishing the neuronal sources of spikes (Tóth et al., 2021), spike collisions are harder to discriminate (Rossant et al. 2016) and electrode drift cannot be corrected perfectly (Georgiadis & SpikeSift, 2025; Steinmetz et al., 2021), and they require a higher signal-to-noise ratio for accurate sorting. In comparison, high-density probes (Jun et al., 2017; Steinmetz et al., 2021) allow for identification of spikes along multiple adjacent sites resulting in a multi-channel waveform with spatial information which allows for better separation (Jia et al., 2019; Ye et al., 2024) between spikes of different neurons and even cell type identification (Ye et al., 2024). Drift correction can be more easily handled in high-density probes due to the coverage making drift appear as a spatial shift (Steinmetz et al., 2018). Moreover, high-density probes allow for the recording of thousands of neurons simultaneously enabling the analysis of neural dynamics (Steinmetz et al., 2018). The number of neurons recorded has seen an exponential increase since the 1950 s (Pachitariu et al., 2016). As recording hardware advances (Jun et al., 2017; Steinmetz et al., 2021), analysis methods must as well to be capable of dealing with the large volume of data that can be obtained. Manifold feature extraction can yield embeddings robust to perturbations (Belkin & Niyogi, 2003) and offer separability in the new feature space. Moreover, manifold techniques have been designed to handle large volumes of data by employing sparse neighbourhood graphs and optimisation for scalability (Amid & TriMap, 2022; McInnes et al., 2020) making them a viable candidate (Amid & TriMap, 2022; McInnes et al., 2020) for the spike sorting of high-density probes (Steinmetz et al., 2021). Hierarchical (Bhatia et al., 2012; Marcílio-Jr et al., 2025) and multi-view extensions (Busch et al., 2023; Rodosthenous et al., 2021) of manifold learning may be suitable techniques for handling the volume and complexity of data obtained through high-density probes.

## Supplementary Information

Below is the link to the electronic supplementary material.

Supplementary file1 (DOCX 1244 KB)

## Data Availability

The datasets used in this work are openly available and can be found at: • Synthetic datasets (19): -SpikeForest tool (95) (RRID:SCR_021532), https://spikeforest.flatironinstitute.org/studyset/SYNTH_MONOTRODE or -http://bioweb.me/CPGJNM2012-dataset or -https://www.kaggle.com/datasets/ardeleanrichard/simulationsdataset/data • Real datasets (82,83) can be found on -SpikeForest tool (95) (RRID:SCR_021532), spe1/paiRED-Kampff: https://spikeforest.flatironinstitute.org/study/paired_kampff or -CRCNS data repository (RRID:SCR_005608), paired Kampff recordings, spe1: https://crcns.org/data-sets/methods/spe-1/about-spe-1

## Code Availability

The code that supports the findings of this work was written in Python and is openly available at: https://github.com/ArdeleanRichard/Nonlinear-Feature-Extraction-in-SpikeSorting.

The software packages used in this study are:

Python 3.12, The Python Software Foundation, https://www.python.org/, RRID:SCR_008394

Numpy, The NumPy community, https://numpy.org/, RRID:SCR_008633

scikit-learn, The scikit-learn community, https://scikit-learn.org/, RRID:SCR_002577

Pandas, The Pandas community, https://pandas.pydata.org/, RRID:SCR_018214

Matplotlib, The Matplotlib community, https://matplotlib.org/, RRID:SCR_008624

Scipy, The Scipy community, https://scipy.org/, RRID:SCR_008058
