# Supplementary material for: A Study of Non-Linear Manifold Feature Extraction in Spike Sorting
Source: Neuroinformatics. 2025 Oct 2;23(4):48. doi: 10.1007/s12021-025-09744-3 (PMC12491110; doi:10.1007/s12021-025-09744-3)
Supplement: Supplementary file 1 — Supplementary file1 (DOCX 1244 KB) [file 12021_2025_9744_MOESM1_ESM.docx]

# Supplementary Material

Table S1. Parametrization of algorithms.

| **Algorithm** | **Parametrization** |
| --- | --- |
| PCA | n_components=2 |
| MDS | n_components=2 |
| ICA | n_components=2, function=logcosh, tolerance=1e-3 |
| KPCA | n_components=2, kernel=rbf, gamma=0.1 |
| SOM | size=10, sigma=1.0, lr=0.5, epochs=1000 |
| AE | n_components=2, hidden_layers = [70,40,20,10], epochs=100, lr=1e-3, batch=64, activation=tanh |
| LLE | n_components=2, n_neighbors=70 |
| MLLE | n_components=2, n_neighbors=50 |
| Isomap | n_components=2, n_neighbors=100, eigensolver=Arnoldi decomposition, path=Dijkstra |
| Spectral embedding | n_components=2, affinity: nearest_neighbors |
| t-SNE | n_components=2, perplexity=30 |
| Diffusion Map | n_components=2, alpha=0.5, k=50 |
| PHATE | n_components=2 |
| UMAP | n_components=2, n_neighbors=10, min_dist=0.05, metric=Chebyshev |
| Trimap | n_components=2 |


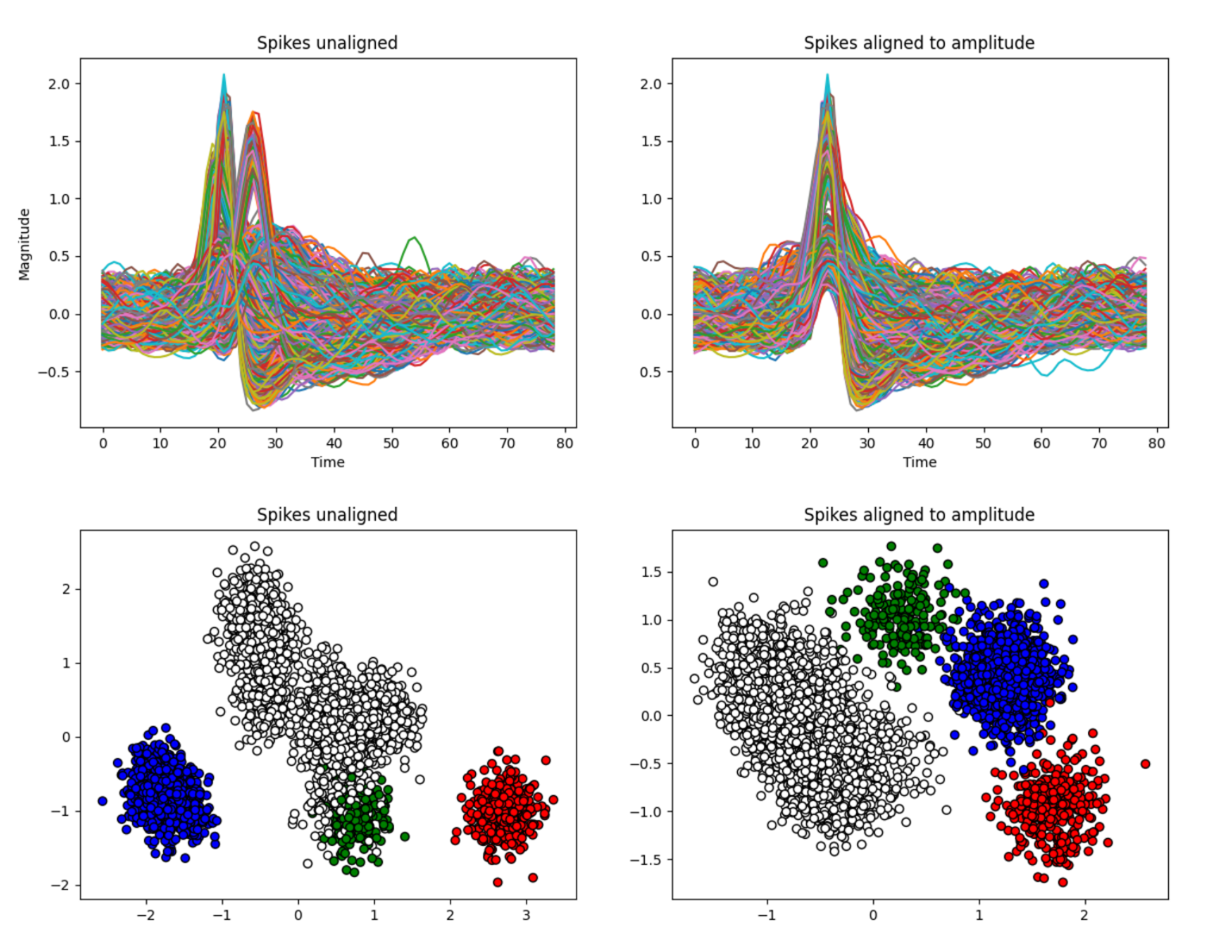


**Fig S1. Impact of alignment on Sim53.** The top row presents the impact of alignment on the spikes themselves, while the bottom row presents the impact of alignment on the PCA features. The white cluster is kept together and the green cluster is now separated from the white cluster.

Table S2. Information about the 95 synthetic datasets (19).

| Simulation number | Number of ground truth clusters /units | Number of spikes |
| --- | --- | --- |
| 1 | 16 | 12012 |
| 2 | 19 | 12784 |
| 3 | 12 | 9277 |
| 4 | 4 | 5127 |
| 5 | 15 | 11758 |
| 6 | 11 | 10186 |
| 7 | 17 | 13999 |
| 8 | 2 | 3494 |
| 9 | 19 | 15653 |
| 10 | 20 | 15149 |
| 11 | 20 | 14982 |
| 12 | 20 | 13488 |
| 13 | 10 | 7792 |
| 14 | 3 | 4507 |
| 15 | 9 | 9683 |
| 16 | 8 | 7556 |
| 17 | 9 | 9098 |
| 18 | 7 | 7004 |
| 19 | 18 | 13807 |
| 20 | 14 | 11186 |
| 21 | 4 | 4293 |
| 22 | 6 | 7101 |
| 23 | 17 | 13374 |
| 24 | 6 | 6302 |
| 26 | 13 | 12313 |
| 28 | 14 | 12081 |
| 29 | 3 | 4177 |
| 30 | 5 | 5210 |
| 31 | 11 | 8224 |
| 32 | 10 | 9078 |
| 33 | 4 | 4169 |
| 34 | 8 | 6851 |
| 35 | 12 | 9481 |
| 36 | 18 | 12377 |
| 37 | 10 | 9954 |
| 38 | 13 | 11148 |
| 39 | 2 | 4719 |
| 40 | 7 | 7360 |
| 41 | 15 | 13347 |
| 42 | 9 | 6746 |
| 43 | 16 | 10871 |
| 45 | 8 | 6666 |
| 46 | 2 | 4202 |
| 47 | 15 | 10977 |
| 48 | 7 | 6823 |
| 49 | 11 | 9496 |
| 50 | 14 | 11211 |
| 51 | 18 | 11746 |
| 52 | 12 | 9255 |
| 53 | 3 | 4490 |
| 54 | 5 | 6923 |
| 55 | 19 | 14117 |
| 56 | 17 | 12784 |
| 57 | 6 | 6301 |
| 58 | 7 | 7860 |
| 59 | 2 | 4176 |
| 60 | 12 | 10158 |
| 61 | 17 | 13280 |
| 62 | 6 | 6649 |
| 63 | 13 | 7634 |
| 64 | 4 | 4890 |
| 65 | 17 | 13523 |
| 66 | 14 | 12572 |
| 67 | 13 | 11377 |
| 68 | 9 | 7170 |
| 69 | 15 | 12051 |
| 70 | 11 | 9395 |
| 71 | 19 | 13459 |
| 72 | 16 | 11183 |
| 73 | 9 | 8436 |
| 74 | 20 | 13065 |
| 75 | 19 | 13908 |
| 76 | 3 | 4791 |
| 77 | 5 | 7084 |
| 78 | 10 | 8518 |
| 79 | 20 | 14536 |
| 80 | 10 | 7757 |
| 81 | 8 | 7937 |
| 82 | 14 | 10430 |
| 83 | 4 | 5206 |
| 84 | 6 | 5809 |
| 85 | 18 | 14512 |
| 86 | 18 | 13847 |
| 87 | 5 | 6671 |
| 88 | 8 | 6414 |
| 89 | 3 | 4573 |
| 90 | 12 | 10565 |
| 91 | 16 | 10821 |
| 92 | 11 | 8184 |
| 93 | 7 | 6113 |
| 94 | 2 | 4990 |
| 95 | 15 | 12462 |


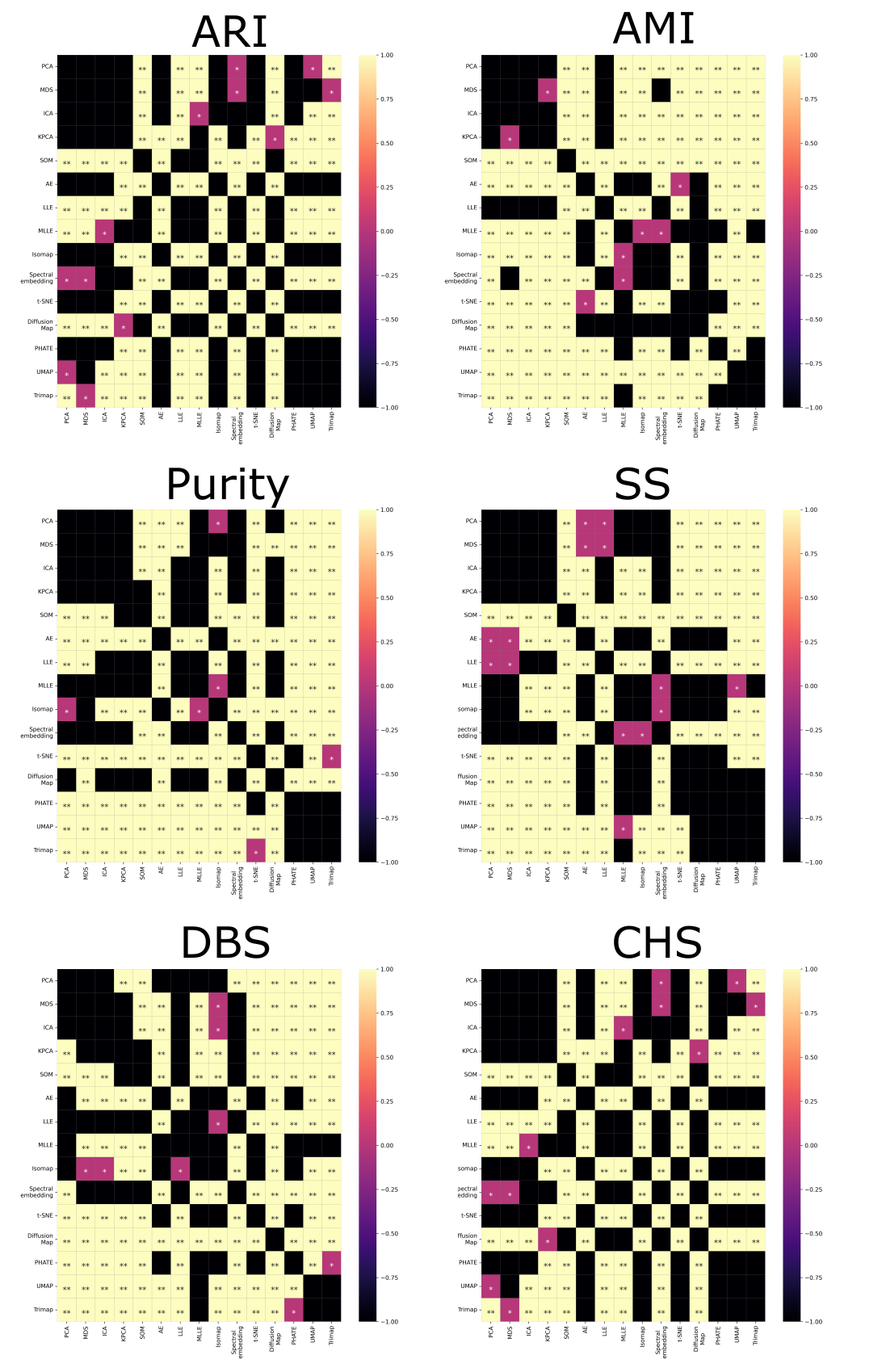


Fig S2 - P value of Mann–Whitney U tests (with a Bonferroni correction) for each of the metric on all 95 simulations (** represents p < 0.01, * represents 0.01<p<0.05, while no text represents 0.05<p).
